# Chlorido{4-ethyl-1-[1-(pyrazin-2-yl)ethyl­idene]thio­semicabazidato-κ*S*}bis­(triphenyl­phosphane-κ*P*)silver(I)

**DOI:** 10.1107/S1600536813003152

**Published:** 2013-02-06

**Authors:** Ramaiyer Venkatraman, Kleopatra D. Ruddock, Himangshu S. Das, Md. Alamgir Hossain, Frank R. Fronczek

**Affiliations:** a Department of Chemistry and Biochemistry, Jackson State University, Jackson, MS 39217-0510, USA; b Department of Civil and Env. Engineering, Jackson State University, Jackson, MS 39217-0510, USA; c Department of Chemistry, Louisiana State University, Baton Rouge, LA 70803, USA

## Abstract

The title compound, [Ag(C_9_H_13_N_5_S)Cl(C_18_H_15_P)_2_], crystallizes with four independent mol­ecules in the asymmetric unit, in each of which the Ag atom is in a distorted tetra­hedral coordination, defined by the chloride ligand, the S atom of the neutral ligand and two P atoms derived from the triphenyl phosphine ligands. The thio­semicarbazone acts as a monodentate ligand through its thione S atom. An intra­molecular N—H⋯Cl hydrogen bond occurs in two of the independent mol­ecules. In the crystal, the mol­ecules are assembled through N—H⋯Cl hydrogen bonds, forming chains along [101].

## Related literature

For general background to thio­semicarbazones, see: Akinchan *et al.* (2002 ▶); Ali & Livingstone (1974 ▶); Bermejo *et al.* (2003 ▶); Blanz & French (1968 ▶); Campbell (1975 ▶); Casas *et al.* (2000 ▶); Grecu & Neamtu (1967 ▶); Hossain *et al.* (2002 ▶); Huheey *et al.* (1993 ▶); Lobana *et al.* (1998 ▶, 2008 ▶); Pellerito & Negy (2002 ▶); Raper (1985 ▶); Venkatraman *et al.* (2009 ▶); Zhou *et al.* (2008 ▶).
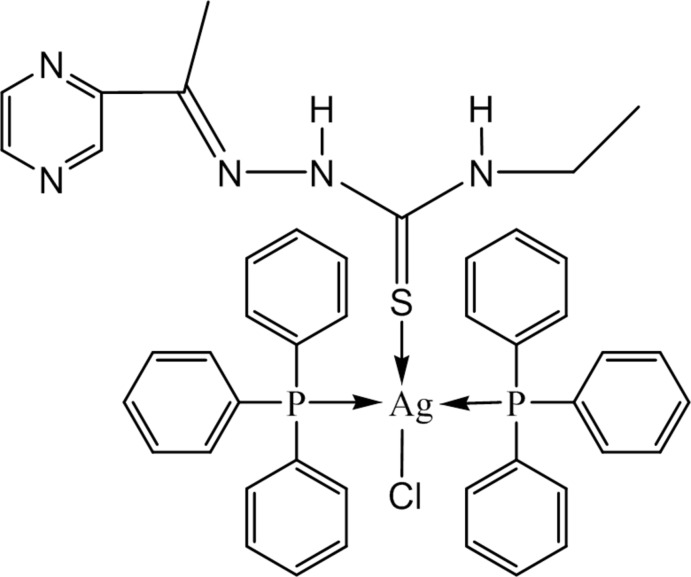


## Experimental

### 

#### Crystal data

[Ag(C_9_H_13_N_5_S)Cl(C_18_H_15_P)_2_]
*M* *_r_* = 891.17
Monoclinic, 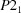

*a* = 12.0640 (5) Å
*b* = 31.810 (2) Å
*c* = 21.9207 (15) Å
β = 98.029 (5)°
*V* = 8329.7 (9) Å^3^
*Z* = 8
Mo *K*α radiation
μ = 0.71 mm^−1^
*T* = 90 K
0.27 × 0.22 × 0.20 mm

#### Data collection

Nonius KappaCCD diffractometer with Oxford Cryostream system
Absorption correction: multi-scan (*DENZO* and *SCALEPACK*; Otwinowski & Minor, 1997 ▶) *T*_min_ = 0.831, *T*_max_ = 0.870
138032 measured reflections
54942 independent reflections
44399 reflections with *I* > 2σ(*I*)
*R*_int_ = 0.055

#### Refinement

*R*[*F* ^2^ > 2σ(*F* ^2^)] = 0.042
*wR*(*F* ^2^) = 0.103
*S* = 1.03
54942 reflections
2007 parameters
9 restraints
H atoms treated by a mixture of independent and constrained refinement
Δρ_max_ = 1.03 e Å^−3^
Δρ_min_ = −0.85 e Å^−3^
Absolute structure: Flack (1983 ▶), 25,519 Friedel pairs
Flack parameter: 0.382 (11)

### 

Data collection: *COLLECT* (Nonius, 2000 ▶); cell refinement: *SCALEPACK* (Otwinowski & Minor, 1997 ▶); data reduction: *DENZO* (Otwinowski & Minor, 1997 ▶) and *SCALEPACK*; program(s) used to solve structure: *SIR97* (Altomare *et al.*, 1999 ▶); program(s) used to refine structure: *SHELXH* (Sheldrick, 2008 ▶); molecular graphics: *ORTEP-3 for Windows* (Farrugia, 2012 ▶); software used to prepare material for publication: *SHELXH*.

## Supplementary Material

Crystal structure: contains datablock(s) global, I. DOI: 10.1107/S1600536813003152/rk2386sup1.cif

Structure factors: contains datablock(s) I. DOI: 10.1107/S1600536813003152/rk2386Isup2.hkl

Additional supplementary materials:  crystallographic information; 3D view; checkCIF report

## Figures and Tables

**Table 1:** Hydrogen-bond geometry (Å, °)

*D*—H⋯*A*	*D*—H	H⋯*A*	*D*⋯*A*	*D*—H⋯*A*
N1—H1*N*⋯Cl1	0.88 (2)	2.54 (2)	3.381 (3)	163 (3)
N3—H3*N*⋯Cl3	0.87 (2)	2.68 (3)	3.406 (3)	142 (3)
N6—H6*N*⋯Cl2	0.88 (2)	2.53 (2)	3.370 (3)	162 (3)
N8—H8*N*⋯Cl4	0.86 (2)	2.68 (3)	3.411 (3)	143 (3)
N11—H11*N*⋯Cl3	0.88 (2)	2.52 (2)	3.363 (3)	161 (3)
N13—H13*N*⋯Cl2	0.88 (2)	2.65 (2)	3.423 (3)	147 (3)
N16—H16*N*⋯Cl4	0.86 (2)	2.50 (2)	3.338 (3)	164 (3)
N18—H18*N*⋯Cl1^i^	0.86 (2)	2.70 (3)	3.425 (3)	143 (3)

Symmetry code: (i) 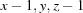.

**Fig. 1.**
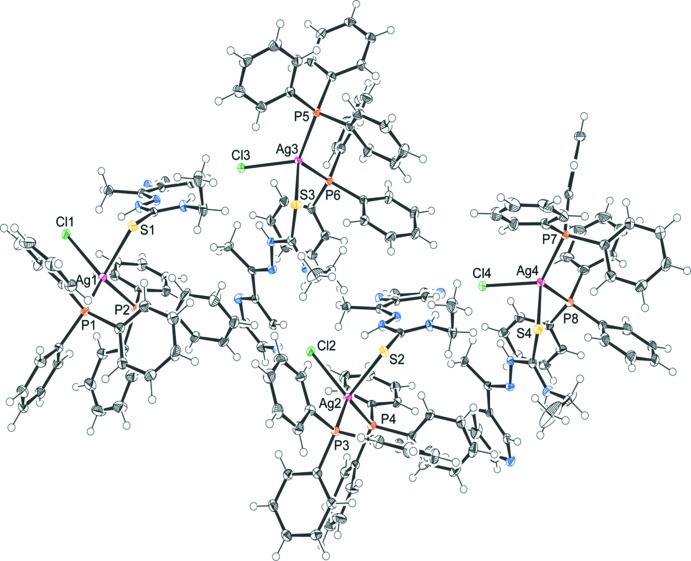
The molecular structure of the title compound. Displacement ellipsoids are drawn at the 50% probability level. H atoms are presented as a small spheres of arbitrary radius. Only base atoms are labeled for clarity.

**Fig. 2.**
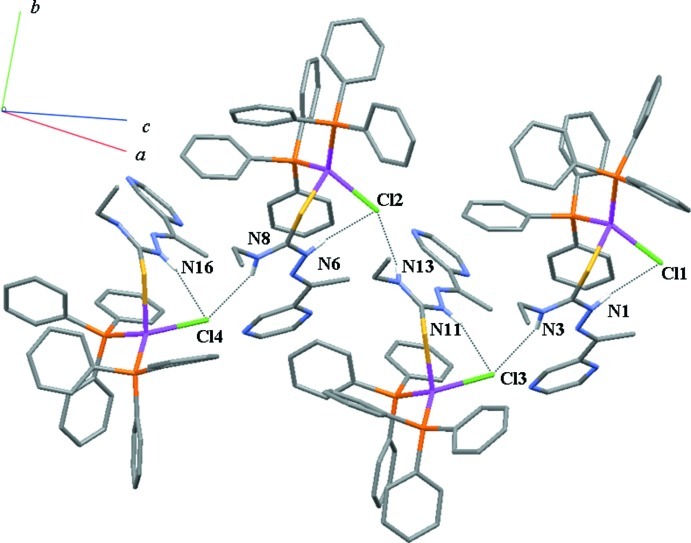
Intra– and inter–molecular hydrogen bonding in the crystal structure of title compound viewed along a axis.

## supplementary crystallographic information

### Comment

Thiosemicarbazones are versatile ligands for several metals due to the keto–enol tautomeric form exhibited by the molecules (Ali & Livingstone, 1974; Campbell, 1975; Pellerito & Negy, 2002; Raper, 1985; Casas *et al.*, 2000; Blanz & French, 1968; Grecu & Neamtu, 1967; Lobana *et al.*, 1998; Bermejo *et al.*, 2003; Akinchan *et al.*, 2002; Zhou *et al.* 2008; Huheey *et al.*, 1993). Among the metals that complex with thiosemicarbazones, Ag(I), and Cu(I) are known to interact with thiosemicarbazones differently and the resulting compounds exhibit variable structural features depending on the conditions of their preparation. Recently, the influence of substituents at the C^2^ carbon of thiosemicarbazones on bonding and nuclearity of silver complexes were investigated (Lobana *et al.*, 2008). Among the ligands that ligate with Ag(I) with pyrazine thiosemicarbazone received less attention.

In continuation of our study on metal complexes with thiosemicarbazones, we report herein a silver complex (Scheme 1) formed by the reaction of 2–acetylpyrazine *N*(4)–ethyl–3–thiosemicarbazone with AgCl in presence of triphenylphosphine as a coligand. The compound crystallizes with four independent molecules in asymmetric unit (Fig. 1). The Ag—S bond distances vary from 2.6816 (8)Å to 2.7412 (9)Å with a mean value of 2.7067 (9)Å. The Ag—S bond distance is smaller than the sum of ionic radii of Ag and S ions (2.78Å). On the other hand, the distances of Ag—P are in the range of 2.4784 (8)Å to 2.4877 (8)Å with a mean value of 2.4835 (8)Å which are smaller than that observed for Ag—S bonds. The average bond distance of Ag—P is comparable to the mononuclear silver complex of thiosemicarbazone (2.4409 (7)Å and 2.4879 (7)Å) reported by Lobana *et al.* (2008). In the asymmetric unit the four Ag—Cl bonds distances are almost similar (2.5900 (8)Å to 2.5932 (8)Å) with a mean value of 2.5921 (8)Å which is shorter than the sum of the ionic radii of silver and chloride (2.75Å). The C—S bond distances in all four units is identical to 1.704 (3)Å which is much longer than the literature value (1.6796 (9)Å) for the free ligand reported by Venkatraman *et al.* (2009). As shown in the Fig. 2, the molecules are linked with intra– and inter–molecular N—H···Cl hydrogen bonding interactions (Hossain *et al.*, 2002).

### Experimental

To a freshly prepared AgCl (0.143 g, 1 mmol) suspended in acetonitrile (20 ml) and was mixed with an acetonitrile solution of 2–acetylpyrazine *N*(4)–ethyl–3–thiosemicarbazone (0.223 g, 1 mmol,) (Venkatraman *et al.*, 2009) and the resulting mixture was stirred overnight. To the white solid formed in acetonitrile, solid triphenylphosphine (0.530 g, 2 mmol) was added in two equal aliquots to obtain a clear solution. The solution was filtered and left for crystallization at room temperature.

### Refinement

H atom of NH group was located in difference syntheses and refined isotropically with *U*_iso_(H) = 1.2*U*_eq_(N). The remaining H atoms were positioned geometrically with C—H = 0.95Å, 0.99Å and 0.98Å for aromatic, methylene and methyl H atoms, respectively, and constrained to ride on their parent atoms with *U*_iso_(H) = *xU*_eq_(C), where *x* = 1.5 for methyl H, and *x* = 1.2 for all other H atoms.

### Crystal data

**Table:**

[Ag(C_9_H_13_N_5_S)Cl(C_18_H_15_P)_2_]	*F*(000) = 3664
*M**_r_* = 891.17	*D*_x_ = 1.421 Mg m^−^^3^
Monoclinic, *P*2_1_	Mo *K*α radiation, λ = 0.71073 Å
Hall symbol: P 2yb	Cell parameters from 28230 reflections
*a* = 12.0640 (5) Å	θ = 2.5–32.5°
*b* = 31.810 (2) Å	µ = 0.71 mm^−^^1^
*c* = 21.9207 (15) Å	*T* = 90 K
β = 98.029 (5)°	Block, colourless
*V* = 8329.7 (9) Å^3^	0.27 × 0.22 × 0.20 mm
*Z* = 8	

### Data collection

**Table:**

Nonius KappaCCD diffractometer with Oxford Cryostream system	54942 independent reflections
Radiation source: fine–focus sealed tube	44399 reflections with *I* > 2σ(*I*)
Graphite monochromator	*R*_int_ = 0.055
ω– and φ–scans	θ_max_ = 32.6°, θ_min_ = 2.5°
Absorption correction: multi-scan (*DENZO* and *SCALEPACK*; Otwinowski & Minor, 1997)	*h* = −18→18
*T*_min_ = 0.831, *T*_max_ = 0.870	*k* = −46→48
138032 measured reflections	*l* = −32→32

### Refinement

**Table:**

Refinement on *F*^2^	Secondary atom site location: difference Fourier map
Least-squares matrix: full	Hydrogen site location: inferred from neighbouring sites
*R*[*F*^2^ > 2σ(*F*^2^)] = 0.042	H atoms treated by a mixture of independent and constrained refinement
*wR*(*F*^2^) = 0.103	*w* = 1/[σ^2^(*F*_o_^2^) + (0.0471*P*)^2^ + 3.4676*P*] where *P* = (*F*_o_^2^ + 2*F*_c_^2^)/3
*S* = 1.03	(Δ/σ)_max_ = 0.002
54942 reflections	Δρ_max_ = 1.03 e Å^−^^3^
2007 parameters	Δρ_min_ = −0.85 e Å^−^^3^
9 restraints	Absolute structure: Flack (1983), 25,519 Friedel pairs
Primary atom site location: structure-invariant direct methods	Flack parameter: 0.382 (11)

### Special details

**Table:**

Geometry. All s.u.'s (except the s.u. in the dihedral angle between two l.s. planes) are estimated using the full covariance matrix. The cell s.u.'s are taken into account individually in the estimation of s.u.'s in distances, angles and torsion angles; correlations between s.u.'s in cell parameters are only used when they are defined by crystal symmetry. An approximate (isotropic) treatment of cell s.u.'s is used for estimating s.u.'s involving l.s. planes.
Refinement. Refinement of *F*^2^ against ALL reflections. The weighted *R*–factor *wR* and goodness of fit *S* are based on *F*^2^, conventional *R*–factors *R* are based on *F*, with *F* set to zero for negative *F*^2^. The threshold expression of *F*^2^ > σ(*F*^2^) is used only for calculating *R*–factors(gt) *etc*. and is not relevant to the choice of reflections for refinement. *R*–factors based on *F*^2^ are statistically about twice as large as those based on *F*, and *R*-factors based on ALL data will be even larger.

### Fractional atomic coordinates and isotropic or equivalent isotropic displacement parameters (Å^2^)

**Table:**

	*x*	*y*	*z*	*U*_iso_*/*U*_eq_	
Ag1	0.723632 (16)	0.688036 (7)	0.820344 (9)	0.01593 (5)	
Cl1	0.86926 (6)	0.65407 (2)	0.90391 (3)	0.01736 (14)	
N1	0.7674 (2)	0.57932 (9)	0.80150 (12)	0.0178 (5)	
H1N	0.803 (3)	0.6002 (8)	0.8216 (14)	0.021*	
N2	0.8198 (2)	0.55463 (9)	0.76375 (13)	0.0172 (5)	
N3	0.5999 (2)	0.56061 (9)	0.74491 (12)	0.0185 (5)	
H3N	0.639 (3)	0.5417 (9)	0.7287 (16)	0.022*	
N4	1.0907 (2)	0.52049 (10)	0.74080 (14)	0.0232 (6)	
N5	0.9582 (2)	0.47812 (10)	0.64508 (13)	0.0217 (6)	
P1	0.59953 (6)	0.74435 (3)	0.84917 (4)	0.01490 (15)	
P2	0.78028 (6)	0.69445 (3)	0.71589 (3)	0.01796 (15)	
S1	0.59041 (6)	0.62016 (3)	0.83052 (4)	0.01636 (14)	
C1	0.6544 (2)	0.58431 (10)	0.78917 (13)	0.0147 (5)	
C2	0.9254 (2)	0.54798 (10)	0.77890 (14)	0.0161 (6)	
C3	0.9931 (3)	0.56410 (12)	0.83686 (16)	0.0219 (7)	
H3A	0.9773	0.5940	0.8417	0.033*	
H3B	1.0729	0.5603	0.8344	0.033*	
H3C	0.9732	0.5485	0.8723	0.033*	
C4	0.9783 (2)	0.52177 (10)	0.73496 (15)	0.0162 (6)	
C5	1.1349 (3)	0.49938 (12)	0.69781 (17)	0.0276 (8)	
H5	1.2141	0.4982	0.7004	0.033*	
C6	1.0700 (3)	0.47899 (11)	0.64920 (16)	0.0237 (7)	
H6	1.1056	0.4655	0.6185	0.028*	
C7	0.9139 (3)	0.49924 (11)	0.68781 (15)	0.0182 (6)	
H7	0.8349	0.4991	0.6864	0.022*	
C8	0.4778 (2)	0.56112 (12)	0.72850 (16)	0.0244 (7)	
H8A	0.4581	0.5491	0.6867	0.029*	
H8B	0.4514	0.5906	0.7272	0.029*	
C9	0.4185 (3)	0.53695 (13)	0.77261 (18)	0.0375 (9)	
H9A	0.3375	0.5386	0.7597	0.056*	
H9B	0.4369	0.5489	0.8140	0.056*	
H9C	0.4422	0.5075	0.7731	0.056*	
C10	0.6477 (2)	0.79894 (10)	0.85914 (13)	0.0163 (6)	
C11	0.7392 (2)	0.81151 (9)	0.83082 (13)	0.0205 (6)	
H11	0.7782	0.7916	0.8094	0.025*	
C12	0.7730 (2)	0.85361 (9)	0.83422 (14)	0.0231 (6)	
H12	0.8353	0.8624	0.8153	0.028*	
C13	0.7158 (3)	0.88233 (11)	0.86504 (14)	0.0245 (7)	
H13	0.7375	0.9111	0.8661	0.029*	
C14	0.6270 (3)	0.86977 (9)	0.89457 (14)	0.0259 (6)	
H14	0.5892	0.8897	0.9165	0.031*	
C15	0.5932 (2)	0.82786 (9)	0.89200 (13)	0.0226 (6)	
H15	0.5330	0.8191	0.9127	0.027*	
C16	0.5438 (2)	0.73372 (9)	0.92063 (13)	0.0181 (6)	
C17	0.4305 (3)	0.73699 (9)	0.92650 (14)	0.0255 (6)	
H17	0.3782	0.7452	0.8921	0.031*	
C18	0.3938 (3)	0.72836 (10)	0.98251 (16)	0.0324 (7)	
H18	0.3164	0.7301	0.9861	0.039*	
C19	0.4705 (3)	0.71709 (10)	1.03343 (16)	0.0362 (8)	
H19	0.4456	0.7119	1.0720	0.043*	
C20	0.5821 (4)	0.71342 (13)	1.02790 (18)	0.0390 (9)	
H20	0.6344	0.7059	1.0627	0.047*	
C21	0.6185 (3)	0.72077 (12)	0.97114 (15)	0.0313 (8)	
H21	0.6951	0.7169	0.9670	0.038*	
C22	0.4748 (2)	0.74862 (10)	0.79088 (13)	0.0187 (6)	
C23	0.4369 (3)	0.78667 (10)	0.76388 (14)	0.0283 (7)	
H23	0.4757	0.8120	0.7758	0.034*	
C24	0.3418 (3)	0.78747 (10)	0.71930 (16)	0.0351 (8)	
H24	0.3160	0.8135	0.7013	0.042*	
C25	0.2852 (3)	0.75088 (11)	0.70126 (15)	0.0313 (7)	
H25	0.2206	0.7517	0.6711	0.038*	
C26	0.3230 (3)	0.71324 (10)	0.72730 (15)	0.0275 (7)	
H26	0.2838	0.6880	0.7153	0.033*	
C27	0.4186 (2)	0.71185 (10)	0.77124 (14)	0.0237 (6)	
H27	0.4454	0.6856	0.7878	0.028*	
C28	0.9007 (3)	0.66345 (10)	0.70072 (15)	0.0184 (6)	
C29	0.9094 (3)	0.64519 (11)	0.64350 (15)	0.0203 (6)	
H29	0.8501	0.6484	0.6104	0.024*	
C30	1.0044 (3)	0.62231 (11)	0.63466 (16)	0.0232 (7)	
H30	1.0089	0.6095	0.5959	0.028*	
C31	1.0919 (3)	0.61826 (12)	0.68209 (16)	0.0244 (7)	
H31	1.1575	0.6034	0.6755	0.029*	
C32	1.0845 (3)	0.63581 (13)	0.73915 (17)	0.0260 (7)	
H32	1.1449	0.6329	0.7717	0.031*	
C33	0.9888 (3)	0.65777 (11)	0.74910 (15)	0.0228 (6)	
H33	0.9830	0.6689	0.7887	0.027*	
C34	0.6669 (2)	0.68237 (10)	0.65440 (13)	0.0210 (6)	
C35	0.5753 (3)	0.66037 (11)	0.66935 (14)	0.0266 (7)	
H35	0.5752	0.6511	0.7105	0.032*	
C36	0.4834 (3)	0.65156 (12)	0.62534 (15)	0.0331 (8)	
H36	0.4211	0.6368	0.6368	0.040*	
C37	0.4826 (3)	0.66418 (10)	0.56546 (14)	0.0280 (7)	
H37	0.4201	0.6580	0.5354	0.034*	
C38	0.5734 (3)	0.68602 (10)	0.54892 (13)	0.0258 (6)	
H38	0.5728	0.6949	0.5075	0.031*	
C39	0.6650 (3)	0.69486 (10)	0.59258 (13)	0.0235 (6)	
H39	0.7272	0.7095	0.5807	0.028*	
C40	0.8220 (3)	0.74817 (10)	0.69748 (14)	0.0248 (7)	
C41	0.9313 (3)	0.76130 (11)	0.71415 (16)	0.0302 (7)	
H41	0.9865	0.7417	0.7312	0.036*	
C42	0.9613 (4)	0.80336 (12)	0.70605 (18)	0.0384 (9)	
H42	1.0362	0.8122	0.7186	0.046*	
C43	0.8836 (4)	0.83164 (12)	0.68024 (19)	0.0479 (11)	
H43	0.9046	0.8600	0.6743	0.057*	
C44	0.7730 (4)	0.81873 (11)	0.66248 (19)	0.0486 (10)	
H44	0.7189	0.8382	0.6439	0.058*	
C45	0.7420 (3)	0.77722 (11)	0.67199 (16)	0.0354 (8)	
H45	0.6663	0.7687	0.6611	0.042*	
Ag2	0.224068 (16)	0.688496 (6)	0.323769 (9)	0.01375 (4)	
Cl2	0.36640 (6)	0.65160 (2)	0.40620 (3)	0.01616 (14)	
N6	0.2600 (2)	0.57945 (9)	0.30150 (12)	0.0164 (5)	
H6N	0.297 (2)	0.6001 (8)	0.3217 (14)	0.020*	
N7	0.3116 (2)	0.55402 (9)	0.26416 (13)	0.0161 (5)	
N8	0.0918 (2)	0.56014 (9)	0.24624 (12)	0.0180 (5)	
H8N	0.129 (3)	0.5404 (9)	0.2313 (16)	0.022*	
N9	0.5816 (2)	0.51946 (9)	0.24039 (14)	0.0211 (6)	
N10	0.4469 (2)	0.47841 (10)	0.14338 (13)	0.0218 (6)	
P3	0.09628 (6)	0.74264 (3)	0.35680 (4)	0.01405 (15)	
P4	0.29648 (6)	0.69742 (2)	0.22380 (3)	0.01487 (15)	
S2	0.08349 (6)	0.62324 (3)	0.32583 (4)	0.01625 (14)	
C46	0.1472 (2)	0.58511 (10)	0.28866 (14)	0.0144 (5)	
C47	0.4171 (2)	0.54722 (10)	0.27876 (14)	0.0156 (6)	
C48	0.4871 (2)	0.56431 (11)	0.33545 (15)	0.0188 (6)	
H48A	0.4723	0.5944	0.3391	0.028*	
H48B	0.5665	0.5600	0.3323	0.028*	
H48C	0.4680	0.5496	0.3719	0.028*	
C49	0.4690 (2)	0.52074 (10)	0.23486 (15)	0.0157 (6)	
C50	0.6249 (3)	0.49865 (12)	0.19625 (17)	0.0260 (7)	
H50	0.7040	0.4969	0.1986	0.031*	
C51	0.5589 (3)	0.47956 (11)	0.14714 (16)	0.0233 (7)	
H51	0.5938	0.4670	0.1154	0.028*	
C52	0.4034 (3)	0.49866 (11)	0.18741 (15)	0.0185 (6)	
H52	0.3246	0.4982	0.1868	0.022*	
C53	−0.0295 (2)	0.56230 (11)	0.22756 (15)	0.0228 (6)	
H53A	−0.0479	0.5494	0.1863	0.027*	
H53B	−0.0524	0.5922	0.2242	0.027*	
C54	−0.0952 (3)	0.54061 (12)	0.27121 (17)	0.0332 (8)	
H54A	−0.1753	0.5434	0.2564	0.050*	
H54B	−0.0784	0.5534	0.3121	0.050*	
H54C	−0.0750	0.5108	0.2737	0.050*	
C55	−0.0384 (2)	0.73692 (9)	0.30721 (14)	0.0174 (6)	
C56	−0.0344 (2)	0.72546 (10)	0.24668 (14)	0.0248 (6)	
H56	0.0361	0.7206	0.2334	0.030*	
C57	−0.1311 (2)	0.72104 (11)	0.20526 (14)	0.0272 (7)	
H57	−0.1264	0.7135	0.1638	0.033*	
C58	−0.2340 (2)	0.72749 (11)	0.22384 (14)	0.0266 (7)	
H58	−0.3004	0.7248	0.1952	0.032*	
C59	−0.2400 (2)	0.73806 (10)	0.28518 (14)	0.0274 (7)	
H59	−0.3109	0.7421	0.2985	0.033*	
C60	−0.1427 (2)	0.74268 (9)	0.32665 (13)	0.0217 (6)	
H60	−0.1471	0.7498	0.3683	0.026*	
C61	0.1279 (2)	0.79871 (10)	0.35066 (13)	0.0156 (6)	
C62	0.0583 (2)	0.82645 (9)	0.31367 (13)	0.0181 (5)	
H62	−0.0088	0.8165	0.2903	0.022*	
C63	0.0874 (2)	0.86885 (9)	0.31104 (14)	0.0214 (6)	
H63	0.0398	0.8878	0.2862	0.026*	
C64	0.1865 (2)	0.88337 (10)	0.34501 (13)	0.0211 (6)	
H64	0.2060	0.9123	0.3438	0.025*	
C65	0.2558 (2)	0.85580 (9)	0.38011 (13)	0.0211 (6)	
H65	0.3239	0.8657	0.4025	0.025*	
C66	0.2272 (2)	0.81349 (9)	0.38338 (13)	0.0192 (5)	
H66	0.2758	0.7947	0.4080	0.023*	
C67	0.0663 (2)	0.73918 (10)	0.43656 (13)	0.0177 (6)	
C68	0.0057 (2)	0.77009 (10)	0.46254 (14)	0.0230 (6)	
H68	−0.0214	0.7938	0.4387	0.028*	
C69	−0.0156 (2)	0.76655 (10)	0.52343 (14)	0.0254 (6)	
H69	−0.0585	0.7873	0.5406	0.030*	
C70	0.0269 (3)	0.73231 (11)	0.55850 (15)	0.0296 (7)	
H70	0.0122	0.7294	0.5998	0.035*	
C71	0.0904 (3)	0.70249 (12)	0.53351 (16)	0.0299 (8)	
H71	0.1204	0.6794	0.5580	0.036*	
C72	0.1110 (3)	0.70583 (10)	0.47272 (15)	0.0223 (6)	
H72	0.1555	0.6853	0.4561	0.027*	
C73	0.4135 (2)	0.66460 (10)	0.20850 (14)	0.0164 (6)	
C74	0.5058 (2)	0.66061 (11)	0.25459 (14)	0.0210 (6)	
H74	0.5047	0.6736	0.2935	0.025*	
C75	0.5991 (3)	0.63762 (12)	0.24352 (16)	0.0244 (7)	
H75	0.6627	0.6359	0.2743	0.029*	
C76	0.5992 (3)	0.61724 (11)	0.18774 (16)	0.0229 (7)	
H76	0.6630	0.6015	0.1804	0.028*	
C77	0.5069 (3)	0.61954 (11)	0.14251 (15)	0.0218 (6)	
H77	0.5067	0.6048	0.1048	0.026*	
C78	0.4146 (3)	0.64344 (11)	0.15252 (15)	0.0180 (6)	
H78	0.3518	0.6454	0.1212	0.022*	
C79	0.3445 (2)	0.75107 (10)	0.21133 (13)	0.0180 (6)	
C80	0.4501 (3)	0.76028 (10)	0.19634 (14)	0.0238 (6)	
H80	0.5009	0.7382	0.1911	0.029*	
C81	0.4816 (3)	0.80230 (11)	0.18893 (16)	0.0296 (7)	
H81	0.5544	0.8085	0.1795	0.036*	
C82	0.4080 (3)	0.83442 (11)	0.19526 (16)	0.0336 (8)	
H82	0.4290	0.8627	0.1892	0.040*	
C83	0.3028 (3)	0.82540 (10)	0.21054 (16)	0.0334 (8)	
H83	0.2521	0.8476	0.2152	0.040*	
C84	0.2712 (3)	0.78410 (9)	0.21902 (14)	0.0260 (6)	
H84	0.1994	0.7782	0.2301	0.031*	
C85	0.1917 (2)	0.68908 (10)	0.15654 (12)	0.0168 (5)	
C86	0.1090 (2)	0.65863 (10)	0.15994 (13)	0.0208 (6)	
H86	0.1085	0.6429	0.1968	0.025*	
C87	0.0275 (3)	0.65125 (10)	0.10965 (14)	0.0248 (6)	
H87	−0.0287	0.6307	0.1125	0.030*	
C88	0.0278 (3)	0.67346 (11)	0.05595 (15)	0.0286 (8)	
H88	−0.0277	0.6680	0.0217	0.034*	
C89	0.1085 (3)	0.70359 (10)	0.05165 (13)	0.0277 (7)	
H89	0.1081	0.7190	0.0145	0.033*	
C90	0.1904 (2)	0.71145 (10)	0.10158 (14)	0.0242 (6)	
H90	0.2460	0.7322	0.0983	0.029*	
Ag3	0.473216 (16)	0.435776 (6)	0.576047 (9)	0.01369 (5)	
Cl3	0.61650 (6)	0.47296 (2)	0.65742 (3)	0.01630 (13)	
N11	0.5127 (2)	0.54412 (9)	0.55109 (12)	0.0157 (5)	
H11N	0.547 (2)	0.5309 (10)	0.5837 (11)	0.019*	
N12	0.5656 (2)	0.56962 (9)	0.51289 (12)	0.0163 (5)	
N13	0.3463 (2)	0.56313 (9)	0.49290 (12)	0.0175 (5)	
H13N	0.381 (3)	0.5821 (9)	0.4736 (15)	0.021*	
N14	0.8364 (2)	0.60499 (9)	0.49304 (14)	0.0224 (6)	
N15	0.7056 (2)	0.64570 (10)	0.39430 (12)	0.0211 (6)	
P5	0.34543 (6)	0.38197 (3)	0.61001 (4)	0.01413 (15)	
P6	0.54480 (6)	0.42590 (3)	0.47599 (3)	0.01462 (15)	
S3	0.33439 (6)	0.50170 (3)	0.57482 (4)	0.01697 (15)	
C91	0.3997 (2)	0.53877 (10)	0.53675 (14)	0.0154 (6)	
C92	0.6714 (2)	0.57670 (10)	0.52943 (14)	0.0159 (6)	
C93	0.7392 (3)	0.56075 (12)	0.58698 (16)	0.0228 (7)	
H93A	0.7154	0.5748	0.6227	0.034*	
H93B	0.8186	0.5666	0.5857	0.034*	
H93C	0.7280	0.5304	0.5903	0.034*	
C94	0.7246 (2)	0.60256 (10)	0.48505 (14)	0.0158 (6)	
C95	0.8817 (3)	0.62717 (12)	0.45030 (17)	0.0265 (8)	
H95	0.9608	0.6296	0.4542	0.032*	
C96	0.8178 (3)	0.64632 (12)	0.40133 (17)	0.0241 (7)	
H96	0.8542	0.6604	0.3715	0.029*	
C97	0.6597 (3)	0.62427 (11)	0.43676 (15)	0.0180 (6)	
H97	0.5805	0.6237	0.4343	0.022*	
C98	0.2258 (2)	0.56039 (11)	0.47262 (15)	0.0213 (6)	
H98A	0.2033	0.5304	0.4695	0.026*	
H98B	0.2084	0.5731	0.4311	0.026*	
C99	0.1591 (3)	0.58266 (13)	0.51643 (18)	0.0378 (9)	
H99A	0.0790	0.5798	0.5016	0.057*	
H99B	0.1794	0.6125	0.5186	0.057*	
H99C	0.1756	0.5701	0.5575	0.057*	
C100	0.3150 (2)	0.38481 (9)	0.68954 (13)	0.0173 (6)	
C101	0.2538 (2)	0.35355 (9)	0.71551 (13)	0.0219 (6)	
H101	0.2265	0.3300	0.6913	0.026*	
C102	0.2326 (2)	0.35663 (11)	0.77614 (14)	0.0258 (6)	
H102	0.1904	0.3355	0.7931	0.031*	
C103	0.2737 (3)	0.39084 (10)	0.81157 (14)	0.0282 (7)	
H103	0.2580	0.3937	0.8526	0.034*	
C104	0.3379 (3)	0.42092 (12)	0.78689 (16)	0.0321 (8)	
H104	0.3684	0.4436	0.8119	0.039*	
C105	0.3584 (3)	0.41839 (10)	0.72604 (15)	0.0248 (7)	
H105	0.4017	0.4394	0.7096	0.030*	
C106	0.3779 (2)	0.32625 (10)	0.60382 (14)	0.0154 (6)	
C107	0.4774 (2)	0.31149 (9)	0.63677 (13)	0.0202 (5)	
H107	0.5256	0.3304	0.6614	0.024*	
C108	0.5068 (2)	0.26930 (10)	0.63396 (13)	0.0228 (6)	
H108	0.5756	0.2597	0.6560	0.027*	
C109	0.4364 (3)	0.24128 (10)	0.59929 (14)	0.0214 (6)	
H109	0.4555	0.2123	0.5985	0.026*	
C110	0.3367 (2)	0.25581 (9)	0.56538 (13)	0.0196 (6)	
H110	0.2889	0.2368	0.5408	0.023*	
C111	0.3075 (2)	0.29808 (9)	0.56751 (13)	0.0185 (5)	
H111	0.2400	0.3078	0.5444	0.022*	
C112	0.2114 (2)	0.38766 (9)	0.56068 (13)	0.0165 (5)	
C113	0.1067 (2)	0.38141 (9)	0.57933 (13)	0.0207 (5)	
H113	0.1017	0.3740	0.6208	0.025*	
C114	0.0087 (2)	0.38603 (10)	0.53726 (14)	0.0250 (6)	
H114	−0.0623	0.3818	0.5503	0.030*	
C115	0.0154 (2)	0.39675 (11)	0.47695 (15)	0.0268 (7)	
H115	−0.0510	0.3995	0.4484	0.032*	
C116	0.1180 (3)	0.40352 (11)	0.45798 (14)	0.0287 (7)	
H116	0.1224	0.4112	0.4165	0.034*	
C117	0.2154 (2)	0.39908 (10)	0.49974 (13)	0.0235 (6)	
H117	0.2860	0.4039	0.4864	0.028*	
C118	0.6609 (2)	0.45917 (10)	0.45967 (14)	0.0170 (6)	
C119	0.7531 (2)	0.46313 (11)	0.50541 (15)	0.0207 (6)	
H119	0.7527	0.4499	0.5442	0.025*	
C120	0.8460 (3)	0.48661 (12)	0.49410 (16)	0.0228 (7)	
H120	0.9090	0.4891	0.5252	0.027*	
C121	0.8467 (3)	0.50637 (11)	0.43751 (16)	0.0221 (7)	
H121	0.9106	0.5217	0.4295	0.027*	
C122	0.7533 (3)	0.50349 (11)	0.39270 (15)	0.0219 (6)	
H122	0.7526	0.5176	0.3545	0.026*	
C123	0.6604 (3)	0.47990 (10)	0.40375 (15)	0.0182 (6)	
H123	0.5968	0.4780	0.3730	0.022*	
C124	0.4400 (2)	0.43286 (10)	0.40783 (12)	0.0172 (5)	
C125	0.4387 (2)	0.40877 (10)	0.35524 (13)	0.0227 (6)	
H125	0.4932	0.3874	0.3538	0.027*	
C126	0.35831 (19)	0.41567 (8)	0.30446 (10)	0.0281 (7)	
H126	0.3575	0.3987	0.2687	0.034*	
C127	0.27943 (19)	0.44705 (8)	0.30562 (10)	0.0266 (7)	
H127	0.2253	0.4520	0.2705	0.032*	
C128	0.2796 (3)	0.47113 (11)	0.35795 (15)	0.0271 (7)	
H128	0.2253	0.4926	0.3590	0.032*	
C129	0.3596 (3)	0.46393 (10)	0.40927 (14)	0.0223 (6)	
H129	0.3591	0.4804	0.4454	0.027*	
C130	0.5952 (2)	0.37254 (10)	0.46526 (13)	0.0186 (6)	
C131	0.7004 (3)	0.36319 (11)	0.45056 (15)	0.0241 (7)	
H131	0.7507	0.3854	0.4452	0.029*	
C132	0.7338 (3)	0.32179 (12)	0.44343 (17)	0.0341 (8)	
H132	0.8064	0.3160	0.4334	0.041*	
C133	0.6611 (3)	0.28918 (11)	0.45095 (16)	0.0344 (8)	
H133	0.6835	0.2609	0.4456	0.041*	
C134	0.5567 (3)	0.29753 (10)	0.46617 (15)	0.0321 (7)	
H134	0.5071	0.2751	0.4717	0.039*	
C135	0.5238 (3)	0.33892 (9)	0.47343 (13)	0.0255 (6)	
H135	0.4515	0.3445	0.4841	0.031*	
Ag4	−0.038125 (18)	0.439474 (6)	0.073147 (9)	0.01726 (5)	
Cl4	0.10740 (6)	0.47297 (2)	0.15720 (3)	0.01678 (14)	
N16	0.0143 (2)	0.54561 (9)	0.05256 (13)	0.0156 (5)	
H16N	0.047 (3)	0.5308 (9)	0.0825 (12)	0.019*	
N17	0.0680 (2)	0.57030 (9)	0.01351 (12)	0.0154 (5)	
N18	−0.1502 (2)	0.56879 (10)	−0.00276 (12)	0.0192 (5)	
H18N	−0.112 (3)	0.5859 (10)	−0.0222 (16)	0.023*	
N19	0.3403 (2)	0.59985 (10)	−0.01125 (14)	0.0248 (6)	
N20	0.2088 (2)	0.64353 (10)	−0.10746 (13)	0.0236 (6)	
P7	−0.15849 (6)	0.38179 (3)	0.10226 (4)	0.01484 (15)	
P8	0.01543 (7)	0.43194 (3)	−0.03167 (3)	0.02084 (16)	
S4	−0.16819 (6)	0.50928 (3)	0.08195 (4)	0.01785 (15)	
C136	−0.0996 (2)	0.54359 (10)	0.04060 (14)	0.0160 (6)	
C137	0.1748 (2)	0.57632 (10)	0.02937 (14)	0.0157 (6)	
C138	0.2424 (3)	0.56014 (12)	0.08687 (15)	0.0197 (7)	
H13A	0.2202	0.5748	0.1226	0.030*	
H13B	0.3220	0.5652	0.0851	0.030*	
H13C	0.2293	0.5299	0.0906	0.030*	
C139	0.2285 (2)	0.60097 (10)	−0.01585 (14)	0.0162 (6)	
C140	0.3846 (3)	0.61961 (14)	−0.05631 (18)	0.0346 (9)	
H140	0.4636	0.6195	−0.0552	0.041*	
C141	0.3205 (3)	0.63988 (13)	−0.10371 (18)	0.0310 (8)	
H141	0.3566	0.6519	−0.1354	0.037*	
C142	0.1638 (3)	0.62441 (11)	−0.06249 (14)	0.0182 (6)	
H142	0.0854	0.6267	−0.0619	0.022*	
C143	−0.2710 (3)	0.57430 (14)	−0.01568 (17)	0.0388 (10)	
H14A	−0.3090	0.5475	−0.0087	0.047*	
H14B	−0.2924	0.5825	−0.0593	0.047*	
C144	−0.3074 (4)	0.60825 (19)	0.0264 (2)	0.0759 (19)	
H14C	−0.3887	0.6119	0.0179	0.114*	
H14D	−0.2704	0.6348	0.0189	0.114*	
H14E	−0.2863	0.5999	0.0695	0.114*	
C145	−0.2137 (2)	0.39298 (10)	0.17381 (13)	0.0212 (6)	
C146	−0.3248 (3)	0.38613 (9)	0.18182 (14)	0.0252 (6)	
H146	−0.3767	0.3760	0.1485	0.030*	
C147	−0.3604 (3)	0.39411 (10)	0.23859 (16)	0.0330 (7)	
H147	−0.4362	0.3895	0.2439	0.040*	
C148	−0.2845 (3)	0.40885 (11)	0.28712 (16)	0.0382 (8)	
H148	−0.3082	0.4137	0.3261	0.046*	
C149	−0.1737 (4)	0.41671 (13)	0.27939 (16)	0.0335 (8)	
H149	−0.1223	0.4270	0.3129	0.040*	
C150	−0.1386 (3)	0.40949 (11)	0.22258 (16)	0.0278 (7)	
H150	−0.0638	0.4157	0.2167	0.033*	
C151	−0.2832 (2)	0.37740 (10)	0.04449 (14)	0.0195 (6)	
C152	−0.3356 (2)	0.41434 (9)	0.02134 (13)	0.0217 (6)	
H152	−0.3062	0.4407	0.0360	0.026*	
C153	−0.4306 (3)	0.41300 (11)	−0.02306 (15)	0.0274 (7)	
H153	−0.4657	0.4384	−0.0380	0.033*	
C154	−0.4739 (3)	0.37496 (11)	−0.04535 (15)	0.0297 (7)	
H154	−0.5388	0.3741	−0.0754	0.036*	
C155	−0.4219 (3)	0.33781 (11)	−0.02347 (15)	0.0325 (7)	
H155	−0.4510	0.3115	−0.0388	0.039*	
C156	−0.3271 (3)	0.33913 (10)	0.02085 (14)	0.0265 (6)	
H156	−0.2917	0.3136	0.0352	0.032*	
C157	−0.1113 (2)	0.32737 (10)	0.11259 (14)	0.0170 (6)	
C158	−0.1672 (2)	0.29837 (9)	0.14576 (13)	0.0229 (6)	
H158	−0.2268	0.3075	0.1666	0.027*	
C159	−0.1352 (2)	0.25615 (9)	0.14803 (14)	0.0240 (6)	
H159	−0.1741	0.2365	0.1698	0.029*	
C160	−0.0466 (3)	0.24285 (10)	0.11850 (14)	0.0236 (6)	
H160	−0.0258	0.2140	0.1194	0.028*	
C161	0.0111 (2)	0.27170 (10)	0.08788 (14)	0.0258 (6)	
H161	0.0729	0.2627	0.0687	0.031*	
C162	−0.0205 (2)	0.31398 (9)	0.08476 (14)	0.0224 (6)	
H162	0.0199	0.3336	0.0637	0.027*	
C163	0.1388 (3)	0.46079 (10)	−0.04771 (14)	0.0187 (6)	
C164	0.2273 (3)	0.46542 (10)	−0.00002 (14)	0.0219 (6)	
H164	0.2201	0.4546	0.0397	0.026*	
C165	0.3260 (3)	0.48554 (12)	−0.00943 (16)	0.0249 (7)	
H165	0.3863	0.4878	0.0232	0.030*	
C166	0.3354 (3)	0.50225 (12)	−0.06692 (16)	0.0257 (7)	
H166	0.4022	0.5162	−0.0736	0.031*	
C167	0.2474 (3)	0.49856 (11)	−0.11457 (15)	0.0242 (7)	
H167	0.2540	0.5102	−0.1538	0.029*	
C168	0.1494 (3)	0.47781 (11)	−0.10522 (15)	0.0209 (6)	
H168	0.0896	0.4753	−0.1381	0.025*	
C169	0.0499 (3)	0.37773 (10)	−0.04887 (14)	0.0304 (7)	
C170	−0.0360 (4)	0.34958 (11)	−0.07160 (16)	0.0453 (10)	
H170	−0.1112	0.3591	−0.0804	0.054*	
C171	−0.0100 (5)	0.30719 (12)	−0.08127 (18)	0.0650 (15)	
H171	−0.0672	0.2883	−0.0981	0.078*	
C172	0.0998 (5)	0.29289 (13)	−0.06615 (19)	0.0625 (15)	
H172	0.1168	0.2641	−0.0714	0.075*	
C173	0.1832 (4)	0.32030 (12)	−0.04381 (19)	0.0507 (11)	
H173	0.2580	0.3105	−0.0343	0.061*	
C174	0.1590 (4)	0.36288 (11)	−0.03481 (16)	0.0367 (9)	
H174	0.2174	0.3816	−0.0191	0.044*	
C175	−0.0980 (3)	0.44532 (11)	−0.09379 (13)	0.0245 (7)	
C176	−0.1838 (3)	0.47031 (11)	−0.07890 (14)	0.0286 (7)	
H176	−0.1809	0.4807	−0.0381	0.034*	
C177	−0.2757 (3)	0.48059 (11)	−0.12342 (15)	0.0324 (7)	
H177	−0.3336	0.4982	−0.1129	0.039*	
C178	−0.2808 (3)	0.46484 (11)	−0.18230 (14)	0.0312 (7)	
H178	−0.3435	0.4709	−0.2123	0.037*	
C179	−0.1953 (3)	0.44043 (11)	−0.19746 (14)	0.0295 (7)	
H179	−0.1987	0.4302	−0.2384	0.035*	
C180	−0.1033 (3)	0.43022 (11)	−0.15419 (14)	0.0302 (7)	
H180	−0.0448	0.4132	−0.1655	0.036*	

### Atomic displacement parameters (Å^2^)

**Table:**

	*U*^11^	*U*^22^	*U*^33^	*U*^12^	*U*^13^	*U*^23^
Ag1	0.01779 (9)	0.01646 (10)	0.01339 (9)	0.00323 (8)	0.00165 (7)	0.00037 (8)
Cl1	0.0175 (3)	0.0188 (4)	0.0146 (3)	0.0009 (3)	−0.0022 (3)	−0.0005 (3)
N1	0.0170 (11)	0.0185 (14)	0.0177 (13)	−0.0021 (10)	0.0020 (10)	−0.0071 (11)
N2	0.0189 (12)	0.0146 (13)	0.0182 (13)	0.0016 (10)	0.0030 (11)	−0.0031 (11)
N3	0.0180 (11)	0.0188 (13)	0.0189 (13)	−0.0015 (10)	0.0033 (10)	−0.0044 (11)
N4	0.0160 (12)	0.0261 (15)	0.0267 (14)	0.0014 (10)	0.0008 (11)	−0.0082 (12)
N5	0.0227 (12)	0.0221 (14)	0.0207 (13)	−0.0002 (11)	0.0043 (11)	−0.0037 (11)
P1	0.0162 (3)	0.0129 (4)	0.0157 (3)	0.0015 (3)	0.0027 (3)	−0.0005 (3)
P2	0.0239 (3)	0.0173 (4)	0.0134 (3)	0.0028 (3)	0.0047 (3)	0.0016 (3)
S1	0.0149 (3)	0.0186 (3)	0.0161 (3)	−0.0003 (3)	0.0040 (3)	−0.0017 (3)
C1	0.0181 (13)	0.0131 (13)	0.0129 (13)	−0.0019 (10)	0.0022 (11)	−0.0010 (11)
C2	0.0158 (12)	0.0159 (15)	0.0157 (14)	−0.0009 (11)	−0.0009 (11)	0.0007 (11)
C3	0.0216 (15)	0.0210 (17)	0.0220 (16)	0.0021 (13)	−0.0007 (13)	−0.0021 (14)
C4	0.0151 (12)	0.0137 (14)	0.0199 (14)	0.0012 (10)	0.0027 (11)	−0.0017 (11)
C5	0.0170 (14)	0.0338 (18)	0.0315 (18)	0.0025 (13)	0.0020 (13)	−0.0143 (15)
C6	0.0223 (14)	0.0251 (16)	0.0238 (16)	0.0025 (12)	0.0039 (12)	−0.0090 (13)
C7	0.0178 (13)	0.0179 (15)	0.0188 (15)	−0.0019 (12)	0.0021 (12)	−0.0017 (12)
C8	0.0163 (13)	0.0357 (17)	0.0212 (14)	−0.0064 (12)	0.0018 (11)	−0.0073 (13)
C9	0.0327 (17)	0.049 (2)	0.0318 (18)	−0.0251 (16)	0.0083 (14)	−0.0037 (16)
C10	0.0194 (13)	0.0148 (14)	0.0144 (13)	0.0006 (10)	0.0008 (10)	−0.0001 (11)
C11	0.0211 (13)	0.0227 (14)	0.0177 (13)	−0.0011 (11)	0.0024 (11)	0.0014 (11)
C12	0.0239 (14)	0.0249 (15)	0.0199 (14)	−0.0057 (11)	0.0011 (11)	0.0011 (11)
C13	0.0319 (16)	0.0192 (15)	0.0216 (15)	−0.0076 (12)	0.0014 (12)	−0.0014 (12)
C14	0.0322 (15)	0.0191 (14)	0.0263 (15)	0.0031 (12)	0.0043 (12)	−0.0040 (12)
C15	0.0234 (13)	0.0211 (14)	0.0240 (14)	0.0000 (11)	0.0062 (11)	−0.0002 (12)
C16	0.0238 (13)	0.0117 (13)	0.0202 (14)	0.0026 (10)	0.0078 (11)	0.0018 (11)
C17	0.0290 (15)	0.0228 (14)	0.0252 (15)	0.0012 (12)	0.0061 (12)	0.0026 (12)
C18	0.0385 (18)	0.0292 (17)	0.0337 (18)	0.0024 (14)	0.0202 (15)	0.0028 (14)
C19	0.062 (2)	0.0206 (15)	0.0308 (18)	0.0096 (16)	0.0247 (17)	0.0053 (13)
C20	0.052 (2)	0.045 (2)	0.0203 (17)	0.0109 (18)	0.0056 (16)	0.0058 (16)
C21	0.0311 (16)	0.045 (2)	0.0185 (15)	0.0074 (15)	0.0041 (13)	0.0009 (15)
C22	0.0177 (12)	0.0222 (14)	0.0163 (13)	0.0036 (11)	0.0025 (10)	0.0004 (11)
C23	0.0335 (16)	0.0195 (14)	0.0278 (16)	0.0002 (12)	−0.0097 (13)	−0.0017 (12)
C24	0.0394 (18)	0.0255 (16)	0.0344 (18)	0.0061 (14)	−0.0158 (14)	0.0023 (14)
C25	0.0253 (15)	0.0385 (19)	0.0270 (17)	0.0040 (14)	−0.0068 (12)	−0.0057 (14)
C26	0.0284 (15)	0.0246 (16)	0.0280 (17)	−0.0037 (13)	−0.0012 (13)	−0.0007 (13)
C27	0.0210 (13)	0.0223 (14)	0.0273 (16)	0.0000 (11)	0.0019 (12)	0.0038 (12)
C28	0.0223 (13)	0.0150 (14)	0.0189 (14)	−0.0012 (11)	0.0067 (11)	0.0008 (11)
C29	0.0203 (14)	0.0238 (16)	0.0173 (14)	−0.0022 (12)	0.0045 (11)	0.0000 (12)
C30	0.0248 (14)	0.0258 (16)	0.0205 (14)	0.0007 (12)	0.0085 (12)	−0.0039 (12)
C31	0.0210 (14)	0.0283 (17)	0.0251 (16)	−0.0001 (13)	0.0077 (13)	−0.0014 (13)
C32	0.0227 (15)	0.0327 (19)	0.0222 (16)	0.0005 (13)	0.0021 (13)	0.0006 (14)
C33	0.0280 (15)	0.0209 (15)	0.0195 (14)	−0.0033 (12)	0.0036 (12)	−0.0023 (12)
C34	0.0284 (14)	0.0210 (16)	0.0136 (12)	0.0077 (12)	0.0031 (10)	0.0033 (11)
C35	0.0302 (15)	0.0346 (17)	0.0139 (13)	−0.0010 (13)	−0.0010 (12)	0.0062 (12)
C36	0.0292 (16)	0.046 (2)	0.0228 (16)	−0.0017 (15)	−0.0006 (13)	0.0036 (15)
C37	0.0366 (17)	0.0274 (16)	0.0182 (14)	0.0045 (13)	−0.0022 (13)	−0.0033 (12)
C38	0.0416 (17)	0.0234 (15)	0.0117 (12)	0.0056 (14)	0.0011 (11)	0.0019 (12)
C39	0.0340 (15)	0.0231 (15)	0.0135 (12)	0.0002 (12)	0.0040 (11)	0.0006 (11)
C40	0.0419 (17)	0.0172 (14)	0.0164 (14)	0.0029 (12)	0.0080 (13)	0.0026 (11)
C41	0.0439 (19)	0.0209 (16)	0.0247 (17)	−0.0061 (14)	0.0010 (14)	0.0021 (13)
C42	0.058 (2)	0.0249 (18)	0.0312 (19)	−0.0120 (16)	0.0024 (16)	0.0002 (15)
C43	0.087 (3)	0.0208 (17)	0.036 (2)	−0.0072 (19)	0.010 (2)	0.0055 (16)
C44	0.081 (3)	0.0213 (17)	0.040 (2)	0.0112 (18)	−0.003 (2)	0.0015 (16)
C45	0.0469 (19)	0.0273 (16)	0.0301 (17)	0.0072 (15)	−0.0009 (15)	0.0011 (14)
Ag2	0.01437 (9)	0.01432 (10)	0.01253 (10)	0.00230 (8)	0.00178 (7)	0.00075 (8)
Cl2	0.0160 (3)	0.0171 (3)	0.0142 (3)	0.0014 (3)	−0.0021 (2)	0.0004 (3)
N6	0.0168 (11)	0.0171 (13)	0.0149 (12)	0.0008 (10)	0.0014 (10)	−0.0046 (10)
N7	0.0173 (11)	0.0145 (13)	0.0171 (13)	0.0021 (10)	0.0040 (10)	−0.0014 (10)
N8	0.0177 (11)	0.0178 (12)	0.0185 (12)	−0.0010 (10)	0.0022 (10)	−0.0054 (10)
N9	0.0174 (11)	0.0211 (13)	0.0245 (14)	0.0025 (10)	0.0024 (10)	−0.0042 (11)
N10	0.0246 (13)	0.0214 (14)	0.0199 (13)	0.0051 (11)	0.0053 (11)	−0.0022 (11)
P3	0.0139 (3)	0.0138 (4)	0.0148 (3)	0.0011 (3)	0.0029 (3)	0.0005 (3)
P4	0.0161 (3)	0.0169 (4)	0.0121 (3)	0.0012 (3)	0.0035 (2)	0.0012 (3)
S2	0.0127 (3)	0.0180 (3)	0.0184 (3)	−0.0007 (2)	0.0035 (3)	−0.0040 (3)
C46	0.0157 (12)	0.0131 (13)	0.0145 (13)	−0.0018 (10)	0.0023 (11)	0.0000 (11)
C47	0.0175 (13)	0.0165 (15)	0.0122 (13)	−0.0009 (11)	−0.0003 (11)	−0.0006 (11)
C48	0.0168 (13)	0.0191 (16)	0.0198 (15)	0.0027 (12)	−0.0002 (12)	−0.0023 (13)
C49	0.0159 (12)	0.0125 (13)	0.0188 (14)	0.0007 (10)	0.0030 (11)	0.0000 (11)
C50	0.0201 (14)	0.0274 (17)	0.0313 (18)	0.0031 (13)	0.0058 (13)	−0.0062 (14)
C51	0.0247 (15)	0.0238 (16)	0.0221 (15)	0.0041 (12)	0.0055 (12)	−0.0066 (13)
C52	0.0191 (13)	0.0184 (15)	0.0179 (14)	0.0038 (12)	0.0017 (11)	−0.0011 (12)
C53	0.0164 (12)	0.0296 (16)	0.0217 (14)	−0.0039 (11)	−0.0003 (11)	−0.0046 (12)
C54	0.0277 (16)	0.040 (2)	0.0324 (18)	−0.0145 (14)	0.0041 (14)	0.0004 (15)
C55	0.0143 (12)	0.0169 (14)	0.0207 (14)	0.0003 (10)	0.0014 (10)	−0.0008 (11)
C56	0.0217 (14)	0.0298 (16)	0.0229 (15)	0.0001 (12)	0.0038 (11)	−0.0046 (13)
C57	0.0219 (14)	0.0407 (19)	0.0186 (14)	0.0027 (13)	0.0011 (11)	−0.0027 (13)
C58	0.0183 (13)	0.0361 (18)	0.0240 (15)	−0.0009 (13)	−0.0018 (12)	−0.0029 (13)
C59	0.0165 (13)	0.0364 (18)	0.0302 (16)	0.0010 (12)	0.0060 (12)	−0.0013 (14)
C60	0.0198 (13)	0.0268 (15)	0.0191 (13)	0.0020 (11)	0.0043 (10)	−0.0015 (12)
C61	0.0167 (12)	0.0173 (14)	0.0143 (12)	0.0003 (10)	0.0073 (10)	−0.0029 (11)
C62	0.0166 (12)	0.0177 (13)	0.0203 (13)	0.0003 (10)	0.0037 (10)	−0.0013 (10)
C63	0.0217 (14)	0.0197 (14)	0.0230 (14)	0.0044 (11)	0.0036 (11)	0.0030 (11)
C64	0.0277 (14)	0.0182 (14)	0.0190 (14)	−0.0042 (12)	0.0084 (12)	−0.0024 (12)
C65	0.0229 (13)	0.0219 (14)	0.0189 (13)	−0.0064 (11)	0.0043 (11)	−0.0027 (11)
C66	0.0200 (13)	0.0206 (13)	0.0170 (13)	0.0006 (10)	0.0023 (10)	0.0004 (11)
C67	0.0186 (13)	0.0178 (14)	0.0178 (13)	0.0008 (11)	0.0068 (11)	0.0000 (11)
C68	0.0224 (13)	0.0250 (15)	0.0217 (14)	0.0023 (11)	0.0031 (11)	−0.0025 (12)
C69	0.0245 (14)	0.0316 (16)	0.0209 (14)	0.0002 (12)	0.0066 (11)	−0.0077 (12)
C70	0.0361 (17)	0.0336 (18)	0.0204 (15)	−0.0071 (14)	0.0092 (13)	0.0000 (13)
C71	0.047 (2)	0.0216 (16)	0.0229 (16)	−0.0007 (14)	0.0095 (15)	0.0050 (13)
C72	0.0305 (15)	0.0185 (15)	0.0183 (14)	0.0003 (12)	0.0048 (12)	0.0028 (11)
C73	0.0185 (12)	0.0151 (13)	0.0159 (13)	0.0002 (10)	0.0042 (10)	0.0016 (11)
C74	0.0226 (14)	0.0252 (16)	0.0145 (13)	0.0031 (12)	0.0006 (11)	−0.0004 (12)
C75	0.0210 (14)	0.0313 (18)	0.0203 (15)	0.0046 (13)	0.0010 (12)	0.0022 (13)
C76	0.0206 (14)	0.0257 (16)	0.0236 (15)	0.0046 (12)	0.0068 (12)	0.0017 (13)
C77	0.0241 (14)	0.0232 (15)	0.0194 (14)	0.0026 (12)	0.0076 (12)	−0.0033 (12)
C78	0.0167 (12)	0.0225 (15)	0.0150 (13)	0.0004 (11)	0.0029 (10)	0.0020 (11)
C79	0.0217 (13)	0.0179 (13)	0.0140 (12)	−0.0027 (11)	0.0007 (10)	0.0010 (10)
C80	0.0250 (14)	0.0251 (16)	0.0201 (14)	−0.0030 (12)	−0.0009 (11)	0.0009 (12)
C81	0.0266 (15)	0.0302 (17)	0.0316 (17)	−0.0097 (13)	0.0027 (13)	0.0051 (14)
C82	0.055 (2)	0.0216 (16)	0.0242 (16)	−0.0104 (15)	0.0056 (15)	0.0041 (13)
C83	0.053 (2)	0.0186 (15)	0.0311 (17)	0.0067 (14)	0.0128 (16)	0.0019 (13)
C84	0.0323 (16)	0.0197 (14)	0.0281 (16)	0.0016 (12)	0.0119 (13)	0.0034 (12)
C85	0.0199 (12)	0.0189 (13)	0.0113 (11)	0.0065 (11)	0.0013 (9)	−0.0004 (10)
C86	0.0267 (14)	0.0200 (14)	0.0144 (13)	0.0018 (12)	−0.0014 (11)	−0.0011 (11)
C87	0.0276 (15)	0.0234 (15)	0.0222 (15)	−0.0021 (12)	−0.0013 (12)	−0.0046 (12)
C88	0.0253 (15)	0.0383 (19)	0.0202 (15)	0.0105 (14)	−0.0038 (12)	−0.0117 (13)
C89	0.0340 (16)	0.0348 (17)	0.0140 (13)	0.0083 (13)	0.0017 (12)	0.0052 (12)
C90	0.0229 (14)	0.0332 (16)	0.0175 (13)	0.0056 (12)	0.0060 (11)	0.0053 (12)
Ag3	0.01369 (9)	0.01420 (12)	0.01306 (9)	−0.00204 (8)	0.00143 (7)	−0.00095 (8)
Cl3	0.0163 (3)	0.0168 (3)	0.0146 (3)	−0.0025 (3)	−0.0019 (3)	0.0001 (3)
N11	0.0162 (11)	0.0147 (12)	0.0159 (12)	−0.0026 (9)	0.0015 (10)	0.0030 (10)
N12	0.0153 (11)	0.0168 (13)	0.0170 (12)	−0.0005 (10)	0.0027 (10)	0.0012 (11)
N13	0.0148 (11)	0.0179 (13)	0.0195 (13)	0.0023 (9)	0.0018 (10)	0.0052 (10)
N14	0.0162 (11)	0.0238 (15)	0.0269 (15)	−0.0017 (10)	0.0018 (11)	0.0066 (12)
N15	0.0203 (12)	0.0250 (15)	0.0177 (13)	0.0005 (10)	0.0009 (10)	0.0048 (11)
P5	0.0130 (3)	0.0142 (4)	0.0153 (3)	−0.0018 (3)	0.0025 (3)	−0.0001 (3)
P6	0.0151 (3)	0.0165 (4)	0.0125 (3)	−0.0019 (3)	0.0028 (2)	−0.0012 (3)
S3	0.0148 (3)	0.0170 (4)	0.0196 (4)	0.0010 (3)	0.0042 (3)	0.0038 (3)
C91	0.0148 (12)	0.0154 (14)	0.0167 (14)	−0.0001 (11)	0.0046 (11)	−0.0021 (11)
C92	0.0189 (13)	0.0110 (13)	0.0180 (14)	−0.0029 (11)	0.0032 (11)	0.0003 (11)
C93	0.0203 (14)	0.0250 (18)	0.0215 (16)	−0.0033 (13)	−0.0023 (13)	0.0100 (14)
C94	0.0180 (13)	0.0150 (14)	0.0142 (13)	−0.0015 (11)	0.0012 (11)	−0.0022 (11)
C95	0.0177 (14)	0.0287 (18)	0.0324 (18)	−0.0020 (13)	0.0006 (13)	0.0110 (15)
C96	0.0195 (14)	0.0270 (18)	0.0270 (17)	−0.0009 (12)	0.0074 (13)	0.0054 (14)
C97	0.0175 (13)	0.0194 (15)	0.0163 (14)	−0.0007 (11)	−0.0004 (11)	0.0009 (12)
C98	0.0186 (13)	0.0244 (16)	0.0204 (14)	0.0041 (11)	0.0010 (11)	0.0048 (12)
C99	0.0298 (17)	0.044 (2)	0.040 (2)	0.0198 (16)	0.0078 (15)	0.0089 (18)
C100	0.0196 (13)	0.0171 (13)	0.0157 (13)	0.0030 (11)	0.0045 (11)	−0.0002 (11)
C101	0.0215 (13)	0.0232 (14)	0.0216 (14)	−0.0022 (11)	0.0046 (11)	−0.0001 (11)
C102	0.0215 (14)	0.0344 (17)	0.0227 (15)	0.0004 (12)	0.0074 (11)	0.0077 (13)
C103	0.0374 (17)	0.0312 (17)	0.0184 (14)	0.0066 (14)	0.0126 (13)	0.0015 (12)
C104	0.051 (2)	0.0247 (17)	0.0219 (16)	−0.0029 (15)	0.0095 (15)	−0.0040 (13)
C105	0.0341 (16)	0.0200 (15)	0.0218 (15)	−0.0058 (12)	0.0092 (13)	−0.0006 (12)
C106	0.0164 (12)	0.0141 (13)	0.0168 (13)	−0.0002 (10)	0.0060 (10)	−0.0014 (11)
C107	0.0208 (13)	0.0232 (14)	0.0162 (13)	−0.0006 (11)	0.0011 (10)	−0.0013 (11)
C108	0.0235 (13)	0.0264 (15)	0.0179 (13)	0.0039 (12)	0.0012 (11)	0.0026 (12)
C109	0.0306 (15)	0.0140 (13)	0.0218 (14)	0.0032 (12)	0.0115 (12)	0.0012 (11)
C110	0.0241 (13)	0.0162 (13)	0.0197 (13)	−0.0005 (11)	0.0077 (11)	−0.0023 (11)
C111	0.0162 (12)	0.0198 (13)	0.0195 (13)	−0.0011 (10)	0.0030 (10)	−0.0023 (11)
C112	0.0145 (11)	0.0156 (13)	0.0190 (13)	−0.0010 (10)	0.0004 (10)	0.0000 (10)
C113	0.0179 (12)	0.0217 (13)	0.0229 (14)	−0.0012 (11)	0.0045 (10)	0.0050 (11)
C114	0.0154 (13)	0.0308 (16)	0.0292 (15)	0.0008 (11)	0.0048 (11)	0.0032 (13)
C115	0.0198 (14)	0.0330 (17)	0.0260 (16)	0.0016 (12)	−0.0028 (12)	0.0003 (13)
C116	0.0261 (15)	0.0401 (19)	0.0186 (14)	−0.0029 (14)	−0.0011 (12)	0.0042 (13)
C117	0.0163 (13)	0.0332 (16)	0.0210 (14)	−0.0032 (12)	0.0024 (11)	0.0026 (12)
C118	0.0164 (12)	0.0185 (14)	0.0165 (13)	−0.0009 (11)	0.0038 (10)	−0.0046 (11)
C119	0.0233 (14)	0.0226 (16)	0.0164 (14)	−0.0038 (12)	0.0037 (11)	−0.0018 (12)
C120	0.0211 (14)	0.0270 (17)	0.0193 (15)	−0.0048 (12)	0.0000 (12)	−0.0007 (13)
C121	0.0180 (13)	0.0238 (16)	0.0254 (16)	−0.0052 (12)	0.0060 (12)	−0.0024 (13)
C122	0.0219 (14)	0.0235 (16)	0.0212 (15)	−0.0033 (12)	0.0056 (12)	0.0023 (12)
C123	0.0172 (13)	0.0185 (15)	0.0192 (14)	−0.0014 (11)	0.0031 (11)	0.0010 (11)
C124	0.0169 (11)	0.0207 (14)	0.0142 (11)	−0.0069 (11)	0.0033 (9)	−0.0006 (11)
C125	0.0194 (13)	0.0315 (16)	0.0168 (13)	−0.0005 (11)	0.0008 (10)	−0.0035 (12)
C126	0.0296 (15)	0.0371 (17)	0.0165 (13)	−0.0058 (13)	−0.0007 (11)	−0.0037 (12)
C127	0.0262 (14)	0.0346 (18)	0.0165 (13)	−0.0097 (13)	−0.0053 (11)	0.0073 (13)
C128	0.0244 (14)	0.0298 (16)	0.0255 (16)	−0.0001 (13)	−0.0022 (12)	0.0082 (13)
C129	0.0281 (15)	0.0206 (15)	0.0175 (14)	−0.0001 (12)	0.0003 (12)	0.0005 (11)
C130	0.0249 (14)	0.0187 (14)	0.0127 (12)	−0.0008 (11)	0.0042 (11)	−0.0021 (11)
C131	0.0208 (14)	0.0238 (16)	0.0269 (16)	0.0015 (12)	0.0003 (12)	−0.0045 (13)
C132	0.0346 (18)	0.034 (2)	0.0329 (19)	0.0137 (15)	0.0011 (15)	−0.0061 (15)
C133	0.052 (2)	0.0227 (16)	0.0290 (18)	0.0115 (15)	0.0079 (16)	0.0024 (14)
C134	0.055 (2)	0.0170 (14)	0.0257 (16)	−0.0024 (14)	0.0108 (15)	−0.0011 (12)
C135	0.0345 (16)	0.0216 (14)	0.0214 (14)	−0.0052 (12)	0.0074 (12)	−0.0013 (12)
Ag4	0.02132 (10)	0.01748 (13)	0.01248 (10)	−0.00586 (9)	0.00067 (8)	0.00058 (8)
Cl4	0.0177 (3)	0.0165 (3)	0.0150 (3)	−0.0013 (3)	−0.0017 (2)	−0.0006 (3)
N16	0.0125 (11)	0.0154 (13)	0.0188 (13)	−0.0024 (9)	0.0018 (10)	0.0033 (10)
N17	0.0164 (11)	0.0153 (13)	0.0148 (12)	−0.0001 (10)	0.0029 (10)	0.0010 (10)
N18	0.0154 (11)	0.0245 (14)	0.0176 (12)	0.0005 (10)	0.0022 (10)	0.0051 (11)
N19	0.0164 (12)	0.0342 (16)	0.0237 (14)	−0.0041 (11)	0.0024 (11)	0.0047 (12)
N20	0.0252 (13)	0.0274 (15)	0.0175 (13)	−0.0065 (11)	−0.0001 (11)	0.0032 (11)
P7	0.0158 (3)	0.0142 (4)	0.0147 (3)	−0.0013 (3)	0.0029 (3)	0.0003 (3)
P8	0.0333 (4)	0.0179 (4)	0.0114 (3)	−0.0063 (3)	0.0035 (3)	−0.0004 (3)
S4	0.0154 (3)	0.0226 (4)	0.0156 (3)	−0.0037 (3)	0.0024 (3)	0.0026 (3)
C136	0.0156 (12)	0.0177 (14)	0.0148 (13)	0.0000 (11)	0.0023 (11)	−0.0020 (11)
C137	0.0165 (13)	0.0138 (14)	0.0167 (14)	−0.0014 (11)	0.0022 (11)	−0.0002 (11)
C138	0.0172 (13)	0.0221 (17)	0.0186 (15)	−0.0034 (12)	−0.0015 (12)	0.0037 (13)
C139	0.0182 (13)	0.0167 (14)	0.0137 (13)	−0.0017 (11)	0.0017 (11)	−0.0029 (11)
C140	0.0189 (15)	0.056 (3)	0.0295 (18)	−0.0103 (15)	0.0058 (14)	0.0049 (17)
C141	0.0245 (16)	0.043 (2)	0.0264 (17)	−0.0117 (15)	0.0076 (14)	0.0044 (15)
C142	0.0179 (13)	0.0202 (16)	0.0163 (14)	−0.0042 (12)	0.0012 (11)	−0.0020 (12)
C143	0.0184 (14)	0.068 (3)	0.0289 (18)	0.0080 (16)	0.0013 (13)	0.0262 (18)
C144	0.054 (3)	0.141 (5)	0.035 (2)	0.068 (3)	0.013 (2)	0.009 (3)
C145	0.0284 (15)	0.0180 (15)	0.0186 (14)	−0.0018 (11)	0.0075 (12)	−0.0017 (11)
C146	0.0303 (15)	0.0194 (14)	0.0280 (15)	−0.0043 (12)	0.0113 (12)	−0.0035 (12)
C147	0.0444 (19)	0.0216 (15)	0.0378 (18)	−0.0027 (14)	0.0225 (16)	−0.0033 (13)
C148	0.068 (2)	0.0283 (18)	0.0233 (16)	−0.0047 (17)	0.0242 (17)	−0.0056 (14)
C149	0.054 (2)	0.0306 (18)	0.0165 (15)	−0.0041 (16)	0.0069 (15)	−0.0040 (13)
C150	0.0311 (16)	0.0317 (18)	0.0206 (15)	−0.0050 (13)	0.0034 (13)	−0.0019 (13)
C151	0.0183 (12)	0.0213 (15)	0.0189 (14)	−0.0028 (11)	0.0030 (10)	0.0032 (12)
C152	0.0218 (13)	0.0204 (14)	0.0233 (14)	0.0030 (11)	0.0043 (11)	−0.0012 (11)
C153	0.0237 (15)	0.0301 (17)	0.0273 (16)	0.0052 (13)	−0.0005 (12)	0.0046 (13)
C154	0.0232 (14)	0.0369 (18)	0.0264 (16)	−0.0040 (13)	−0.0053 (12)	0.0020 (14)
C155	0.0368 (17)	0.0284 (16)	0.0295 (17)	−0.0066 (14)	−0.0060 (14)	−0.0018 (13)
C156	0.0303 (15)	0.0227 (15)	0.0239 (15)	−0.0021 (12)	−0.0052 (12)	−0.0012 (12)
C157	0.0162 (12)	0.0156 (14)	0.0187 (14)	−0.0011 (10)	0.0008 (10)	−0.0022 (11)
C158	0.0233 (13)	0.0212 (14)	0.0242 (14)	0.0002 (11)	0.0035 (11)	0.0054 (12)
C159	0.0251 (14)	0.0199 (14)	0.0266 (15)	−0.0008 (11)	0.0023 (11)	0.0058 (12)
C160	0.0293 (15)	0.0194 (14)	0.0206 (14)	0.0054 (12)	−0.0020 (12)	0.0015 (12)
C161	0.0254 (14)	0.0298 (16)	0.0219 (15)	0.0047 (12)	0.0025 (11)	−0.0027 (12)
C162	0.0226 (13)	0.0210 (14)	0.0234 (15)	−0.0021 (11)	0.0032 (11)	−0.0001 (11)
C163	0.0274 (14)	0.0168 (14)	0.0130 (13)	0.0014 (11)	0.0065 (11)	−0.0003 (11)
C164	0.0319 (15)	0.0202 (15)	0.0146 (13)	0.0003 (12)	0.0065 (12)	−0.0005 (11)
C165	0.0299 (16)	0.0259 (17)	0.0193 (15)	0.0032 (13)	0.0047 (13)	0.0026 (12)
C166	0.0218 (14)	0.0315 (18)	0.0251 (16)	0.0043 (13)	0.0074 (13)	0.0025 (14)
C167	0.0271 (15)	0.0273 (16)	0.0196 (14)	0.0047 (13)	0.0079 (12)	0.0063 (13)
C168	0.0259 (15)	0.0194 (15)	0.0182 (14)	0.0022 (12)	0.0057 (12)	0.0044 (12)
C169	0.058 (2)	0.0185 (14)	0.0149 (14)	−0.0035 (15)	0.0044 (14)	0.0015 (12)
C170	0.077 (3)	0.0235 (16)	0.0295 (18)	−0.0127 (17)	−0.0135 (18)	0.0062 (14)
C171	0.134 (4)	0.0237 (18)	0.029 (2)	−0.019 (2)	−0.018 (2)	−0.0024 (15)
C172	0.133 (5)	0.0194 (18)	0.033 (2)	0.002 (2)	0.002 (3)	−0.0023 (16)
C173	0.091 (3)	0.0270 (19)	0.038 (2)	0.015 (2)	0.022 (2)	0.0006 (16)
C174	0.068 (3)	0.0170 (15)	0.0266 (17)	−0.0010 (16)	0.0137 (17)	0.0021 (13)
C175	0.0344 (15)	0.0246 (16)	0.0137 (12)	−0.0121 (14)	0.0004 (11)	0.0028 (12)
C176	0.0351 (17)	0.0351 (18)	0.0154 (14)	−0.0094 (14)	0.0037 (12)	−0.0015 (13)
C177	0.0383 (18)	0.0376 (18)	0.0202 (15)	−0.0051 (15)	0.0000 (13)	−0.0003 (13)
C178	0.0386 (18)	0.0322 (18)	0.0198 (15)	−0.0118 (14)	−0.0064 (13)	0.0040 (13)
C179	0.0510 (19)	0.0249 (17)	0.0105 (12)	−0.0118 (15)	−0.0027 (12)	−0.0007 (12)
C180	0.0490 (19)	0.0231 (15)	0.0182 (14)	−0.0037 (14)	0.0034 (13)	−0.0022 (12)

### Geometric parameters (Å, º)

**Table:**

Ag1—P1	2.4727 (8)	Ag3—P5	2.4862 (8)
Ag1—P2	2.4877 (8)	Ag3—P6	2.4869 (8)
Ag1—Cl1	2.5905 (8)	Ag3—Cl3	2.5900 (8)
Ag1—S1	2.7192 (9)	Ag3—S3	2.6816 (8)
N1—N2	1.359 (3)	N11—C91	1.366 (4)
N1—C1	1.361 (4)	N11—N12	1.384 (4)
N1—H1N	0.875 (17)	N11—H11N	0.882 (17)
N2—C2	1.289 (4)	N12—C92	1.296 (4)
N3—C1	1.328 (4)	N13—C91	1.329 (4)
N3—C8	1.468 (4)	N13—C98	1.463 (4)
N3—H3N	0.871 (17)	N13—H13N	0.878 (17)
N4—C5	1.328 (4)	N14—C94	1.338 (4)
N4—C4	1.345 (4)	N14—C95	1.348 (4)
N5—C7	1.325 (4)	N15—C97	1.335 (4)
N5—C6	1.339 (4)	N15—C96	1.340 (4)
P1—C16	1.821 (3)	P5—C106	1.824 (3)
P1—C10	1.835 (3)	P5—C112	1.825 (3)
P1—C22	1.838 (3)	P5—C100	1.833 (3)
P2—C34	1.822 (3)	P6—C130	1.829 (3)
P2—C28	1.824 (3)	P6—C118	1.830 (3)
P2—C40	1.842 (3)	P6—C124	1.831 (3)
S1—C1	1.707 (3)	S3—C91	1.700 (3)
C2—C4	1.483 (4)	C92—C94	1.486 (4)
C2—C3	1.501 (5)	C92—C93	1.493 (5)
C3—H3A	0.9800	C93—H93A	0.9800
C3—H3B	0.9800	C93—H93B	0.9800
C3—H3C	0.9800	C93—H93C	0.9800
C4—C7	1.400 (4)	C94—C97	1.406 (4)
C5—C6	1.391 (5)	C95—C96	1.373 (5)
C5—H5	0.9500	C95—H95	0.9500
C6—H6	0.9500	C96—H96	0.9500
C7—H7	0.9500	C97—H97	0.9500
C8—C9	1.494 (5)	C98—C99	1.512 (5)
C8—H8A	0.9900	C98—H98A	0.9900
C8—H8B	0.9900	C98—H98B	0.9900
C9—H9A	0.9800	C99—H99A	0.9800
C9—H9B	0.9800	C99—H99B	0.9800
C9—H9C	0.9800	C99—H99C	0.9800
C10—C15	1.389 (4)	C100—C105	1.392 (4)
C10—C11	1.398 (4)	C100—C101	1.405 (4)
C11—C12	1.399 (4)	C101—C102	1.391 (4)
C11—H11	0.9500	C101—H101	0.9500
C12—C13	1.377 (4)	C102—C103	1.388 (4)
C12—H12	0.9500	C102—H102	0.9500
C13—C14	1.386 (4)	C103—C104	1.387 (5)
C13—H13	0.9500	C103—H103	0.9500
C14—C15	1.393 (4)	C104—C105	1.392 (4)
C14—H14	0.9500	C104—H104	0.9500
C15—H15	0.9500	C105—H105	0.9500
C16—C21	1.389 (4)	C106—C107	1.393 (4)
C16—C17	1.394 (4)	C106—C111	1.403 (4)
C17—C18	1.389 (4)	C107—C108	1.392 (4)
C17—H17	0.9500	C107—H107	0.9500
C18—C19	1.393 (5)	C108—C109	1.383 (4)
C18—H18	0.9500	C108—H108	0.9500
C19—C20	1.373 (5)	C109—C110	1.401 (4)
C19—H19	0.9500	C109—H109	0.9500
C20—C21	1.396 (5)	C110—C111	1.392 (4)
C20—H20	0.9500	C110—H110	0.9500
C21—H21	0.9500	C111—H111	0.9500
C22—C27	1.390 (4)	C112—C117	1.392 (4)
C22—C23	1.396 (4)	C112—C113	1.395 (4)
C23—C24	1.400 (4)	C113—C114	1.401 (4)
C23—H23	0.9500	C113—H113	0.9500
C24—C25	1.379 (5)	C114—C115	1.379 (4)
C24—H24	0.9500	C114—H114	0.9500
C25—C26	1.377 (5)	C115—C116	1.377 (4)
C25—H25	0.9500	C115—H115	0.9500
C26—C27	1.396 (4)	C116—C117	1.392 (4)
C26—H26	0.9500	C116—H116	0.9500
C27—H27	0.9500	C117—H117	0.9500
C28—C29	1.399 (4)	C118—C123	1.391 (4)
C28—C33	1.404 (5)	C118—C119	1.395 (4)
C29—C30	1.394 (4)	C119—C120	1.398 (4)
C29—H29	0.9500	C119—H119	0.9500
C30—C31	1.381 (5)	C120—C121	1.392 (5)
C30—H30	0.9500	C120—H120	0.9500
C31—C32	1.384 (5)	C121—C122	1.390 (5)
C31—H31	0.9500	C121—H121	0.9500
C32—C33	1.393 (5)	C122—C123	1.397 (4)
C32—H32	0.9500	C122—H122	0.9500
C33—H33	0.9500	C123—H123	0.9500
C34—C35	1.385 (4)	C124—C125	1.383 (4)
C34—C39	1.409 (4)	C124—C129	1.388 (4)
C35—C36	1.393 (5)	C125—C126	1.388 (3)
C35—H35	0.9500	C125—H125	0.9500
C36—C37	1.371 (4)	C126—C127	1.382 (4)
C36—H36	0.9500	C126—H126	0.9500
C37—C38	1.387 (5)	C127—C128	1.379 (4)
C37—H37	0.9500	C127—H127	0.9500
C38—C39	1.386 (4)	C128—C129	1.395 (4)
C38—H38	0.9500	C128—H128	0.9500
C39—H39	0.9500	C129—H129	0.9500
C40—C41	1.383 (5)	C130—C131	1.385 (4)
C40—C45	1.395 (5)	C130—C135	1.400 (4)
C41—C42	1.404 (5)	C131—C132	1.392 (5)
C41—H41	0.9500	C131—H131	0.9500
C42—C43	1.363 (6)	C132—C133	1.383 (5)
C42—H42	0.9500	C132—H132	0.9500
C43—C44	1.398 (6)	C133—C134	1.373 (5)
C43—H43	0.9500	C133—H133	0.9500
C44—C45	1.396 (5)	C134—C135	1.391 (4)
C44—H44	0.9500	C134—H134	0.9500
C45—H45	0.9500	C135—H135	0.9500
Ag2—P3	2.4858 (8)	Ag4—P7	2.4784 (8)
Ag2—P4	2.4861 (8)	Ag4—P8	2.4838 (8)
Ag2—Cl2	2.5932 (8)	Ag4—Cl4	2.5901 (8)
Ag2—S2	2.6848 (8)	Ag4—S4	2.7412 (9)
N6—N7	1.362 (3)	N16—C136	1.364 (4)
N6—C46	1.363 (4)	N16—N17	1.387 (3)
N6—H6N	0.877 (17)	N16—H16N	0.860 (17)
N7—C47	1.287 (4)	N17—C137	1.300 (4)
N8—C46	1.330 (4)	N18—C136	1.325 (4)
N8—C53	1.465 (4)	N18—C143	1.456 (4)
N8—H8N	0.861 (17)	N18—H18N	0.860 (17)
N9—C50	1.337 (4)	N19—C139	1.339 (4)
N9—C49	1.347 (4)	N19—C140	1.343 (4)
N10—C52	1.328 (4)	N20—C142	1.336 (4)
N10—C51	1.343 (4)	N20—C141	1.344 (4)
P3—C61	1.833 (3)	P7—C145	1.823 (3)
P3—C55	1.834 (3)	P7—C157	1.826 (3)
P3—C67	1.838 (3)	P7—C151	1.832 (3)
P4—C85	1.822 (3)	P8—C163	1.825 (3)
P4—C73	1.824 (3)	P8—C169	1.825 (3)
P4—C79	1.835 (3)	P8—C175	1.840 (3)
S2—C46	1.703 (3)	S4—C136	1.705 (3)
C47—C49	1.482 (4)	C137—C139	1.482 (4)
C47—C48	1.503 (4)	C137—C138	1.494 (4)
C48—H48A	0.9800	C138—H13A	0.9800
C48—H48B	0.9800	C138—H13B	0.9800
C48—H48C	0.9800	C138—H13C	0.9800
C49—C52	1.404 (4)	C139—C142	1.410 (5)
C50—C51	1.386 (5)	C140—C141	1.367 (5)
C50—H50	0.9500	C140—H140	0.9500
C51—H51	0.9500	C141—H141	0.9500
C52—H52	0.9500	C142—H142	0.9500
C53—C54	1.494 (4)	C143—C144	1.524 (6)
C53—H53A	0.9900	C143—H14A	0.9900
C53—H53B	0.9900	C143—H14B	0.9900
C54—H54A	0.9800	C144—H14C	0.9800
C54—H54B	0.9800	C144—H14D	0.9800
C54—H54C	0.9800	C144—H14E	0.9800
C55—C56	1.383 (4)	C145—C146	1.393 (4)
C55—C60	1.396 (4)	C145—C150	1.403 (5)
C56—C57	1.382 (4)	C146—C147	1.395 (4)
C56—H56	0.9500	C146—H146	0.9500
C57—C58	1.375 (4)	C147—C148	1.385 (5)
C57—H57	0.9500	C147—H147	0.9500
C58—C59	1.398 (4)	C148—C149	1.392 (5)
C58—H58	0.9500	C148—H148	0.9500
C59—C60	1.389 (4)	C149—C150	1.389 (5)
C59—H59	0.9500	C149—H149	0.9500
C60—H60	0.9500	C150—H150	0.9500
C61—C66	1.389 (4)	C151—C152	1.396 (4)
C61—C62	1.397 (4)	C151—C156	1.398 (4)
C62—C63	1.397 (4)	C152—C153	1.397 (4)
C62—H62	0.9500	C152—H152	0.9500
C63—C64	1.396 (4)	C153—C154	1.380 (5)
C63—H63	0.9500	C153—H153	0.9500
C64—C65	1.371 (4)	C154—C155	1.391 (5)
C64—H64	0.9500	C154—H154	0.9500
C65—C66	1.393 (4)	C155—C156	1.394 (4)
C65—H65	0.9500	C155—H155	0.9500
C66—H66	0.9500	C156—H156	0.9500
C67—C72	1.387 (4)	C157—C162	1.393 (4)
C67—C68	1.393 (4)	C157—C158	1.404 (4)
C68—C69	1.399 (4)	C158—C159	1.397 (4)
C68—H68	0.9500	C158—H158	0.9500
C69—C70	1.389 (4)	C159—C160	1.391 (4)
C69—H69	0.9500	C159—H159	0.9500
C70—C71	1.380 (5)	C160—C161	1.381 (4)
C70—H70	0.9500	C160—H160	0.9500
C71—C72	1.393 (4)	C161—C162	1.397 (4)
C71—H71	0.9500	C161—H161	0.9500
C72—H72	0.9500	C162—H162	0.9500
C73—C74	1.401 (4)	C163—C164	1.393 (4)
C73—C78	1.401 (4)	C163—C168	1.394 (4)
C74—C75	1.392 (4)	C164—C165	1.392 (5)
C74—H74	0.9500	C164—H164	0.9500
C75—C76	1.384 (5)	C165—C166	1.387 (5)
C75—H75	0.9500	C165—H165	0.9500
C76—C77	1.385 (5)	C166—C167	1.386 (5)
C76—H76	0.9500	C166—H166	0.9500
C77—C78	1.392 (4)	C167—C168	1.395 (4)
C77—H77	0.9500	C167—H167	0.9500
C78—H78	0.9500	C168—H168	0.9500
C79—C80	1.391 (4)	C169—C174	1.392 (5)
C79—C84	1.399 (4)	C169—C170	1.407 (5)
C80—C81	1.405 (5)	C170—C171	1.407 (5)
C80—H80	0.9500	C170—H170	0.9500
C81—C82	1.374 (5)	C171—C172	1.396 (7)
C81—H81	0.9500	C171—H171	0.9500
C82—C83	1.386 (5)	C172—C173	1.369 (7)
C82—H82	0.9500	C172—H172	0.9500
C83—C84	1.388 (4)	C173—C174	1.405 (5)
C83—H83	0.9500	C173—H173	0.9500
C84—H84	0.9500	C174—H174	0.9500
C85—C90	1.397 (4)	C175—C176	1.380 (5)
C85—C86	1.400 (4)	C175—C180	1.401 (4)
C86—C87	1.391 (4)	C176—C177	1.409 (5)
C86—H86	0.9500	C176—H176	0.9500
C87—C88	1.373 (5)	C177—C178	1.378 (5)
C87—H87	0.9500	C177—H177	0.9500
C88—C89	1.379 (5)	C178—C179	1.369 (5)
C88—H88	0.9500	C178—H178	0.9500
C89—C90	1.391 (4)	C179—C180	1.394 (4)
C89—H89	0.9500	C179—H179	0.9500
C90—H90	0.9500	C180—H180	0.9500
			
P1—Ag1—P2	115.46 (3)	P5—Ag3—P6	118.91 (3)
P1—Ag1—Cl1	119.77 (3)	P5—Ag3—Cl3	119.36 (3)
P2—Ag1—Cl1	116.02 (3)	P6—Ag3—Cl3	112.62 (2)
P1—Ag1—S1	99.69 (3)	P5—Ag3—S3	97.41 (2)
P2—Ag1—S1	112.46 (3)	P6—Ag3—S3	112.54 (3)
Cl1—Ag1—S1	87.76 (3)	Cl3—Ag3—S3	90.32 (3)
N2—N1—C1	119.0 (3)	C91—N11—N12	117.6 (3)
N2—N1—H1N	121 (2)	C91—N11—H11N	118 (2)
C1—N1—H1N	114 (2)	N12—N11—H11N	125 (2)
C2—N2—N1	117.7 (3)	C92—N12—N11	116.9 (3)
C1—N3—C8	123.4 (3)	C91—N13—C98	123.1 (3)
C1—N3—H3N	117 (2)	C91—N13—H13N	122 (2)
C8—N3—H3N	119 (2)	C98—N13—H13N	115 (2)
C5—N4—C4	116.6 (3)	C94—N14—C95	116.0 (3)
C7—N5—C6	116.3 (3)	C97—N15—C96	116.0 (3)
C16—P1—C10	102.86 (13)	C106—P5—C112	103.53 (14)
C16—P1—C22	104.30 (13)	C106—P5—C100	101.08 (13)
C10—P1—C22	103.17 (14)	C112—P5—C100	106.26 (13)
C16—P1—Ag1	113.27 (10)	C106—P5—Ag3	119.87 (9)
C10—P1—Ag1	121.53 (10)	C112—P5—Ag3	106.84 (10)
C22—P1—Ag1	110.00 (10)	C100—P5—Ag3	117.75 (10)
C34—P2—C28	106.60 (14)	C130—P6—C118	103.54 (14)
C34—P2—C40	103.49 (15)	C130—P6—C124	102.20 (14)
C28—P2—C40	102.28 (15)	C118—P6—C124	103.33 (13)
C34—P2—Ag1	112.92 (10)	C130—P6—Ag3	113.15 (9)
C28—P2—Ag1	116.85 (11)	C118—P6—Ag3	117.96 (10)
C40—P2—Ag1	113.34 (10)	C124—P6—Ag3	114.79 (9)
C1—S1—Ag1	99.81 (10)	C91—S3—Ag3	102.46 (10)
N3—C1—N1	117.3 (3)	N13—C91—N11	117.4 (3)
N3—C1—S1	123.6 (2)	N13—C91—S3	123.3 (2)
N1—C1—S1	119.2 (2)	N11—C91—S3	119.3 (2)
N2—C2—C4	115.1 (3)	N12—C92—C94	114.3 (3)
N2—C2—C3	124.3 (3)	N12—C92—C93	125.3 (3)
C4—C2—C3	120.6 (3)	C94—C92—C93	120.4 (3)
C2—C3—H3A	109.5	C92—C93—H93A	109.5
C2—C3—H3B	109.5	C92—C93—H93B	109.5
H3A—C3—H3B	109.5	H93A—C93—H93B	109.5
C2—C3—H3C	109.5	C92—C93—H93C	109.5
H3A—C3—H3C	109.5	H93A—C93—H93C	109.5
H3B—C3—H3C	109.5	H93B—C93—H93C	109.5
N4—C4—C7	120.2 (3)	N14—C94—C97	121.0 (3)
N4—C4—C2	118.3 (3)	N14—C94—C92	117.8 (3)
C7—C4—C2	121.5 (3)	C97—C94—C92	121.3 (3)
N4—C5—C6	122.7 (3)	N14—C95—C96	122.5 (3)
N4—C5—H5	118.7	N14—C95—H95	118.7
C6—C5—H5	118.7	C96—C95—H95	118.7
N5—C6—C5	121.0 (3)	N15—C96—C95	122.0 (3)
N5—C6—H6	119.5	N15—C96—H96	119.0
C5—C6—H6	119.5	C95—C96—H96	119.0
N5—C7—C4	123.0 (3)	N15—C97—C94	122.3 (3)
N5—C7—H7	118.5	N15—C97—H97	118.9
C4—C7—H7	118.5	C94—C97—H97	118.9
N3—C8—C9	113.0 (3)	N13—C98—C99	111.9 (3)
N3—C8—H8A	109.0	N13—C98—H98A	109.2
C9—C8—H8A	109.0	C99—C98—H98A	109.2
N3—C8—H8B	109.0	N13—C98—H98B	109.2
C9—C8—H8B	109.0	C99—C98—H98B	109.2
H8A—C8—H8B	107.8	H98A—C98—H98B	107.9
C8—C9—H9A	109.5	C98—C99—H99A	109.5
C8—C9—H9B	109.5	C98—C99—H99B	109.5
H9A—C9—H9B	109.5	H99A—C99—H99B	109.5
C8—C9—H9C	109.5	C98—C99—H99C	109.5
H9A—C9—H9C	109.5	H99A—C99—H99C	109.5
H9B—C9—H9C	109.5	H99B—C99—H99C	109.5
C15—C10—C11	120.1 (3)	C105—C100—C101	119.1 (3)
C15—C10—P1	121.5 (2)	C105—C100—P5	118.4 (2)
C11—C10—P1	118.3 (2)	C101—C100—P5	122.4 (2)
C10—C11—C12	119.5 (3)	C102—C101—C100	120.9 (3)
C10—C11—H11	120.2	C102—C101—H101	119.5
C12—C11—H11	120.2	C100—C101—H101	119.5
C13—C12—C11	119.9 (3)	C103—C102—C101	119.4 (3)
C13—C12—H12	120.0	C103—C102—H102	120.3
C11—C12—H12	120.0	C101—C102—H102	120.3
C12—C13—C14	120.7 (3)	C104—C103—C102	119.9 (3)
C12—C13—H13	119.6	C104—C103—H103	120.1
C14—C13—H13	119.6	C102—C103—H103	120.1
C13—C14—C15	119.9 (3)	C103—C104—C105	121.1 (3)
C13—C14—H14	120.1	C103—C104—H104	119.4
C15—C14—H14	120.1	C105—C104—H104	119.4
C10—C15—C14	119.8 (3)	C100—C105—C104	119.5 (3)
C10—C15—H15	120.1	C100—C105—H105	120.3
C14—C15—H15	120.1	C104—C105—H105	120.3
C21—C16—C17	118.9 (3)	C107—C106—C111	119.2 (3)
C21—C16—P1	117.7 (2)	C107—C106—P5	117.8 (2)
C17—C16—P1	123.4 (2)	C111—C106—P5	122.9 (2)
C18—C17—C16	120.3 (3)	C108—C107—C106	120.5 (3)
C18—C17—H17	119.9	C108—C107—H107	119.7
C16—C17—H17	119.9	C106—C107—H107	119.7
C17—C18—C19	120.1 (3)	C109—C108—C107	120.3 (3)
C17—C18—H18	120.0	C109—C108—H108	119.8
C19—C18—H18	120.0	C107—C108—H108	119.8
C20—C19—C18	120.0 (3)	C108—C109—C110	119.6 (3)
C20—C19—H19	120.0	C108—C109—H109	120.2
C18—C19—H19	120.0	C110—C109—H109	120.2
C19—C20—C21	119.9 (4)	C111—C110—C109	120.2 (3)
C19—C20—H20	120.0	C111—C110—H110	119.9
C21—C20—H20	120.0	C109—C110—H110	119.9
C16—C21—C20	120.7 (3)	C110—C111—C106	120.0 (3)
C16—C21—H21	119.6	C110—C111—H111	120.0
C20—C21—H21	119.6	C106—C111—H111	120.0
C27—C22—C23	118.8 (3)	C117—C112—C113	118.2 (3)
C27—C22—P1	118.0 (2)	C117—C112—P5	116.6 (2)
C23—C22—P1	123.1 (2)	C113—C112—P5	125.2 (2)
C22—C23—C24	120.0 (3)	C112—C113—C114	120.5 (3)
C22—C23—H23	120.0	C112—C113—H113	119.8
C24—C23—H23	120.0	C114—C113—H113	119.8
C25—C24—C23	120.6 (3)	C115—C114—C113	120.0 (3)
C25—C24—H24	119.7	C115—C114—H114	120.0
C23—C24—H24	119.7	C113—C114—H114	120.0
C26—C25—C24	119.5 (3)	C116—C115—C114	120.3 (3)
C26—C25—H25	120.2	C116—C115—H115	119.9
C24—C25—H25	120.2	C114—C115—H115	119.9
C25—C26—C27	120.5 (3)	C115—C116—C117	119.8 (3)
C25—C26—H26	119.8	C115—C116—H116	120.1
C27—C26—H26	119.8	C117—C116—H116	120.1
C22—C27—C26	120.5 (3)	C112—C117—C116	121.2 (3)
C22—C27—H27	119.8	C112—C117—H117	119.4
C26—C27—H27	119.8	C116—C117—H117	119.4
C29—C28—C33	118.7 (3)	C123—C118—C119	119.7 (3)
C29—C28—P2	123.3 (3)	C123—C118—P6	122.5 (2)
C33—C28—P2	118.0 (2)	C119—C118—P6	117.8 (2)
C30—C29—C28	120.5 (3)	C118—C119—C120	119.9 (3)
C30—C29—H29	119.8	C118—C119—H119	120.0
C28—C29—H29	119.8	C120—C119—H119	120.0
C31—C30—C29	120.1 (3)	C121—C120—C119	120.3 (3)
C31—C30—H30	120.0	C121—C120—H120	119.9
C29—C30—H30	120.0	C119—C120—H120	119.9
C30—C31—C32	120.2 (3)	C122—C121—C120	119.7 (3)
C30—C31—H31	119.9	C122—C121—H121	120.2
C32—C31—H31	119.9	C120—C121—H121	120.2
C31—C32—C33	120.3 (3)	C121—C122—C123	120.2 (3)
C31—C32—H32	119.9	C121—C122—H122	119.9
C33—C32—H32	119.9	C123—C122—H122	119.9
C32—C33—C28	120.2 (3)	C118—C123—C122	120.2 (3)
C32—C33—H33	119.9	C118—C123—H123	119.9
C28—C33—H33	119.9	C122—C123—H123	119.9
C35—C34—C39	117.6 (3)	C125—C124—C129	119.1 (3)
C35—C34—P2	118.2 (2)	C125—C124—P6	122.7 (2)
C39—C34—P2	124.2 (2)	C129—C124—P6	118.2 (2)
C34—C35—C36	121.5 (3)	C124—C125—C126	120.4 (3)
C34—C35—H35	119.3	C124—C125—H125	119.8
C36—C35—H35	119.3	C126—C125—H125	119.8
C37—C36—C35	120.1 (3)	C127—C126—C125	120.36 (16)
C37—C36—H36	119.9	C127—C126—H126	119.8
C35—C36—H36	119.9	C125—C126—H126	119.8
C36—C37—C38	119.8 (3)	C128—C127—C126	119.70 (17)
C36—C37—H37	120.1	C128—C127—H127	120.1
C38—C37—H37	120.1	C126—C127—H127	120.1
C39—C38—C37	120.2 (3)	C127—C128—C129	120.0 (3)
C39—C38—H38	119.9	C127—C128—H128	120.0
C37—C38—H38	119.9	C129—C128—H128	120.0
C38—C39—C34	120.7 (3)	C124—C129—C128	120.4 (3)
C38—C39—H39	119.6	C124—C129—H129	119.8
C34—C39—H39	119.6	C128—C129—H129	119.8
C41—C40—C45	119.1 (3)	C131—C130—C135	117.8 (3)
C41—C40—P2	120.1 (3)	C131—C130—P6	124.1 (2)
C45—C40—P2	120.5 (3)	C135—C130—P6	118.1 (2)
C40—C41—C42	120.5 (4)	C130—C131—C132	121.2 (3)
C40—C41—H41	119.7	C130—C131—H131	119.4
C42—C41—H41	119.7	C132—C131—H131	119.4
C43—C42—C41	120.5 (4)	C133—C132—C131	119.8 (3)
C43—C42—H42	119.8	C133—C132—H132	120.1
C41—C42—H42	119.8	C131—C132—H132	120.1
C42—C43—C44	119.7 (4)	C134—C133—C132	120.2 (3)
C42—C43—H43	120.2	C134—C133—H133	119.9
C44—C43—H43	120.2	C132—C133—H133	119.9
C45—C44—C43	120.1 (4)	C133—C134—C135	119.8 (3)
C45—C44—H44	119.9	C133—C134—H134	120.1
C43—C44—H44	119.9	C135—C134—H134	120.1
C40—C45—C44	120.1 (4)	C134—C135—C130	121.2 (3)
C40—C45—H45	120.0	C134—C135—H135	119.4
C44—C45—H45	120.0	C130—C135—H135	119.4
P3—Ag2—P4	119.09 (3)	P7—Ag4—P8	113.84 (3)
P3—Ag2—Cl2	119.20 (3)	P7—Ag4—Cl4	118.99 (3)
P4—Ag2—Cl2	112.70 (2)	P8—Ag4—Cl4	117.37 (3)
P3—Ag2—S2	96.42 (3)	P7—Ag4—S4	102.70 (2)
P4—Ag2—S2	113.81 (3)	P8—Ag4—S4	111.64 (3)
Cl2—Ag2—S2	89.81 (3)	Cl4—Ag4—S4	87.39 (2)
N7—N6—C46	118.8 (3)	C136—N16—N17	116.9 (3)
N7—N6—H6N	120 (2)	C136—N16—H16N	118 (2)
C46—N6—H6N	115 (2)	N17—N16—H16N	125 (2)
C47—N7—N6	118.0 (3)	C137—N17—N16	116.7 (3)
C46—N8—C53	123.5 (3)	C136—N18—C143	124.1 (3)
C46—N8—H8N	118 (2)	C136—N18—H18N	121 (2)
C53—N8—H8N	119 (2)	C143—N18—H18N	114 (2)
C50—N9—C49	116.2 (3)	C139—N19—C140	115.6 (3)
C52—N10—C51	116.0 (3)	C142—N20—C141	115.0 (3)
C61—P3—C55	103.27 (14)	C145—P7—C157	102.91 (14)
C61—P3—C67	101.48 (13)	C145—P7—C151	104.25 (14)
C55—P3—C67	106.38 (13)	C157—P7—C151	103.01 (14)
C61—P3—Ag2	120.58 (9)	C145—P7—Ag4	112.01 (11)
C55—P3—Ag2	107.03 (10)	C157—P7—Ag4	123.37 (10)
C67—P3—Ag2	116.64 (10)	C151—P7—Ag4	109.43 (10)
C85—P4—C73	103.60 (13)	C163—P8—C169	102.70 (15)
C85—P4—C79	102.28 (14)	C163—P8—C175	106.46 (14)
C73—P4—C79	103.66 (14)	C169—P8—C175	103.47 (16)
C85—P4—Ag2	114.03 (9)	C163—P8—Ag4	117.03 (10)
C73—P4—Ag2	118.23 (10)	C169—P8—Ag4	112.24 (10)
C79—P4—Ag2	113.24 (10)	C175—P8—Ag4	113.55 (10)
C46—S2—Ag2	102.38 (10)	C136—S4—Ag4	98.98 (10)
N8—C46—N6	117.1 (3)	N18—C136—N16	117.4 (3)
N8—C46—S2	122.9 (2)	N18—C136—S4	124.0 (2)
N6—C46—S2	120.0 (2)	N16—C136—S4	118.6 (2)
N7—C47—C49	115.3 (3)	N17—C137—C139	114.1 (3)
N7—C47—C48	124.5 (3)	N17—C137—C138	125.3 (3)
C49—C47—C48	120.2 (3)	C139—C137—C138	120.5 (3)
C47—C48—H48A	109.5	C137—C138—H13A	109.5
C47—C48—H48B	109.5	C137—C138—H13B	109.5
H48A—C48—H48B	109.5	H13A—C138—H13B	109.5
C47—C48—H48C	109.5	C137—C138—H13C	109.5
H48A—C48—H48C	109.5	H13A—C138—H13C	109.5
H48B—C48—H48C	109.5	H13B—C138—H13C	109.5
N9—C49—C52	120.5 (3)	N19—C139—C142	121.3 (3)
N9—C49—C47	118.1 (3)	N19—C139—C137	117.8 (3)
C52—C49—C47	121.3 (3)	C142—C139—C137	121.0 (3)
N9—C50—C51	122.6 (3)	N19—C140—C141	122.6 (3)
N9—C50—H50	118.7	N19—C140—H140	118.7
C51—C50—H50	118.7	C141—C140—H140	118.7
N10—C51—C50	121.6 (3)	N20—C141—C140	122.9 (3)
N10—C51—H51	119.2	N20—C141—H141	118.6
C50—C51—H51	119.2	C140—C141—H141	118.6
N10—C52—C49	122.9 (3)	N20—C142—C139	122.3 (3)
N10—C52—H52	118.6	N20—C142—H142	118.8
C49—C52—H52	118.6	C139—C142—H142	118.8
N8—C53—C54	113.4 (3)	N18—C143—C144	109.4 (3)
N8—C53—H53A	108.9	N18—C143—H14A	109.8
C54—C53—H53A	108.9	C144—C143—H14A	109.8
N8—C53—H53B	108.9	N18—C143—H14B	109.8
C54—C53—H53B	108.9	C144—C143—H14B	109.8
H53A—C53—H53B	107.7	H14A—C143—H14B	108.2
C53—C54—H54A	109.5	C143—C144—H14C	109.5
C53—C54—H54B	109.5	C143—C144—H14D	109.5
H54A—C54—H54B	109.5	H14C—C144—H14D	109.5
C53—C54—H54C	109.5	C143—C144—H14E	109.5
H54A—C54—H54C	109.5	H14C—C144—H14E	109.5
H54B—C54—H54C	109.5	H14D—C144—H14E	109.5
C56—C55—C60	118.8 (3)	C146—C145—C150	119.5 (3)
C56—C55—P3	116.6 (2)	C146—C145—P7	123.5 (2)
C60—C55—P3	124.6 (2)	C150—C145—P7	117.0 (2)
C57—C56—C55	121.2 (3)	C145—C146—C147	120.3 (3)
C57—C56—H56	119.4	C145—C146—H146	119.8
C55—C56—H56	119.4	C147—C146—H146	119.8
C58—C57—C56	120.3 (3)	C148—C147—C146	119.6 (3)
C58—C57—H57	119.8	C148—C147—H147	120.2
C56—C57—H57	119.8	C146—C147—H147	120.2
C57—C58—C59	119.4 (3)	C147—C148—C149	120.7 (3)
C57—C58—H58	120.3	C147—C148—H148	119.7
C59—C58—H58	120.3	C149—C148—H148	119.7
C60—C59—C58	120.2 (3)	C150—C149—C148	119.8 (4)
C60—C59—H59	119.9	C150—C149—H149	120.1
C58—C59—H59	119.9	C148—C149—H149	120.1
C59—C60—C55	120.1 (3)	C149—C150—C145	120.0 (3)
C59—C60—H60	119.9	C149—C150—H150	120.0
C55—C60—H60	119.9	C145—C150—H150	120.0
C66—C61—C62	119.4 (3)	C152—C151—C156	118.0 (3)
C66—C61—P3	117.7 (2)	C152—C151—P7	118.3 (2)
C62—C61—P3	122.9 (2)	C156—C151—P7	123.7 (2)
C61—C62—C63	120.0 (3)	C151—C152—C153	120.9 (3)
C61—C62—H62	120.0	C151—C152—H152	119.6
C63—C62—H62	120.0	C153—C152—H152	119.6
C64—C63—C62	119.9 (3)	C154—C153—C152	120.4 (3)
C64—C63—H63	120.0	C154—C153—H153	119.8
C62—C63—H63	120.0	C152—C153—H153	119.8
C65—C64—C63	119.8 (3)	C153—C154—C155	119.5 (3)
C65—C64—H64	120.1	C153—C154—H154	120.3
C63—C64—H64	120.1	C155—C154—H154	120.3
C64—C65—C66	120.8 (3)	C154—C155—C156	120.1 (3)
C64—C65—H65	119.6	C154—C155—H155	120.0
C66—C65—H65	119.6	C156—C155—H155	120.0
C61—C66—C65	120.1 (3)	C155—C156—C151	121.1 (3)
C61—C66—H66	119.9	C155—C156—H156	119.5
C65—C66—H66	119.9	C151—C156—H156	119.5
C72—C67—C68	119.4 (3)	C162—C157—C158	119.3 (3)
C72—C67—P3	118.5 (2)	C162—C157—P7	119.1 (2)
C68—C67—P3	122.1 (2)	C158—C157—P7	121.5 (2)
C67—C68—C69	120.6 (3)	C159—C158—C157	120.0 (3)
C67—C68—H68	119.7	C159—C158—H158	120.0
C69—C68—H68	119.7	C157—C158—H158	120.0
C70—C69—C68	119.3 (3)	C160—C159—C158	120.2 (3)
C70—C69—H69	120.4	C160—C159—H159	119.9
C68—C69—H69	120.4	C158—C159—H159	119.9
C71—C70—C69	120.1 (3)	C161—C160—C159	119.8 (3)
C71—C70—H70	119.9	C161—C160—H160	120.1
C69—C70—H70	119.9	C159—C160—H160	120.1
C70—C71—C72	120.7 (3)	C160—C161—C162	120.7 (3)
C70—C71—H71	119.7	C160—C161—H161	119.6
C72—C71—H71	119.7	C162—C161—H161	119.6
C67—C72—C71	119.9 (3)	C157—C162—C161	119.9 (3)
C67—C72—H72	120.1	C157—C162—H162	120.0
C71—C72—H72	120.1	C161—C162—H162	120.0
C74—C73—C78	119.0 (3)	C164—C163—C168	118.7 (3)
C74—C73—P4	118.5 (2)	C164—C163—P8	117.9 (2)
C78—C73—P4	122.5 (2)	C168—C163—P8	123.3 (2)
C75—C74—C73	120.2 (3)	C165—C164—C163	121.2 (3)
C75—C74—H74	119.9	C165—C164—H164	119.4
C73—C74—H74	119.9	C163—C164—H164	119.4
C76—C75—C74	120.1 (3)	C166—C165—C164	119.4 (3)
C76—C75—H75	120.0	C166—C165—H165	120.3
C74—C75—H75	120.0	C164—C165—H165	120.3
C75—C76—C77	120.5 (3)	C167—C166—C165	120.1 (3)
C75—C76—H76	119.7	C167—C166—H166	119.9
C77—C76—H76	119.7	C165—C166—H166	119.9
C76—C77—C78	119.8 (3)	C166—C167—C168	120.3 (3)
C76—C77—H77	120.1	C166—C167—H167	119.8
C78—C77—H77	120.1	C168—C167—H167	119.8
C77—C78—C73	120.4 (3)	C163—C168—C167	120.2 (3)
C77—C78—H78	119.8	C163—C168—H168	119.9
C73—C78—H78	119.8	C167—C168—H168	119.9
C80—C79—C84	119.1 (3)	C174—C169—C170	119.3 (3)
C80—C79—P4	123.4 (2)	C174—C169—P8	120.7 (3)
C84—C79—P4	117.5 (2)	C170—C169—P8	119.8 (3)
C79—C80—C81	119.9 (3)	C169—C170—C171	119.6 (4)
C79—C80—H80	120.0	C169—C170—H170	120.2
C81—C80—H80	120.0	C171—C170—H170	120.2
C82—C81—C80	120.4 (3)	C172—C171—C170	120.0 (4)
C82—C81—H81	119.8	C172—C171—H171	120.0
C80—C81—H81	119.8	C170—C171—H171	120.0
C81—C82—C83	119.8 (3)	C173—C172—C171	120.2 (4)
C81—C82—H82	120.1	C173—C172—H172	119.9
C83—C82—H82	120.1	C171—C172—H172	119.9
C82—C83—C84	120.4 (3)	C172—C173—C174	120.5 (5)
C82—C83—H83	119.8	C172—C173—H173	119.7
C84—C83—H83	119.8	C174—C173—H173	119.7
C83—C84—C79	120.3 (3)	C169—C174—C173	120.3 (4)
C83—C84—H84	119.8	C169—C174—H174	119.9
C79—C84—H84	119.8	C173—C174—H174	119.9
C90—C85—C86	118.4 (3)	C176—C175—C180	118.9 (3)
C90—C85—P4	123.1 (2)	C176—C175—P8	117.6 (2)
C86—C85—P4	118.5 (2)	C180—C175—P8	123.4 (3)
C87—C86—C85	120.3 (3)	C175—C176—C177	120.9 (3)
C87—C86—H86	119.8	C175—C176—H176	119.6
C85—C86—H86	119.8	C177—C176—H176	119.6
C88—C87—C86	120.4 (3)	C178—C177—C176	119.5 (3)
C88—C87—H87	119.8	C178—C177—H177	120.2
C86—C87—H87	119.8	C176—C177—H177	120.2
C87—C88—C89	120.2 (3)	C179—C178—C177	119.8 (3)
C87—C88—H88	119.9	C179—C178—H178	120.1
C89—C88—H88	119.9	C177—C178—H178	120.1
C88—C89—C90	120.1 (3)	C178—C179—C180	121.5 (3)
C88—C89—H89	119.9	C178—C179—H179	119.3
C90—C89—H89	119.9	C180—C179—H179	119.3
C89—C90—C85	120.5 (3)	C179—C180—C175	119.4 (3)
C89—C90—H90	119.7	C179—C180—H180	120.3
C85—C90—H90	119.7	C175—C180—H180	120.3
			
C1—N1—N2—C2	−175.1 (3)	C91—N11—N12—C92	175.4 (3)
P2—Ag1—P1—C16	171.02 (11)	P6—Ag3—P5—C106	45.33 (12)
Cl1—Ag1—P1—C16	−42.66 (11)	Cl3—Ag3—P5—C106	−99.22 (12)
S1—Ag1—P1—C16	50.32 (11)	S3—Ag3—P5—C106	166.21 (12)
P2—Ag1—P1—C10	−65.75 (12)	P6—Ag3—P5—C112	−71.79 (10)
Cl1—Ag1—P1—C10	80.56 (12)	Cl3—Ag3—P5—C112	143.66 (10)
S1—Ag1—P1—C10	173.54 (12)	S3—Ag3—P5—C112	49.10 (10)
P2—Ag1—P1—C22	54.75 (11)	P6—Ag3—P5—C100	168.89 (10)
Cl1—Ag1—P1—C22	−158.94 (10)	Cl3—Ag3—P5—C100	24.33 (11)
S1—Ag1—P1—C22	−65.95 (11)	S3—Ag3—P5—C100	−70.23 (11)
P1—Ag1—P2—C34	−72.92 (12)	P5—Ag3—P6—C130	−45.98 (11)
Cl1—Ag1—P2—C34	139.47 (12)	Cl3—Ag3—P6—C130	100.82 (11)
S1—Ag1—P2—C34	40.56 (12)	S3—Ag3—P6—C130	−158.84 (10)
P1—Ag1—P2—C28	162.91 (12)	P5—Ag3—P6—C118	−167.00 (12)
Cl1—Ag1—P2—C28	15.31 (13)	Cl3—Ag3—P6—C118	−20.20 (12)
S1—Ag1—P2—C28	−83.61 (12)	S3—Ag3—P6—C118	80.13 (12)
P1—Ag1—P2—C40	44.35 (12)	P5—Ag3—P6—C124	70.80 (12)
Cl1—Ag1—P2—C40	−103.25 (12)	Cl3—Ag3—P6—C124	−142.39 (11)
S1—Ag1—P2—C40	157.84 (12)	S3—Ag3—P6—C124	−42.06 (12)
P1—Ag1—S1—C1	162.42 (11)	P5—Ag3—S3—C91	−167.61 (11)
P2—Ag1—S1—C1	39.57 (11)	P6—Ag3—S3—C91	−42.04 (12)
Cl1—Ag1—S1—C1	−77.75 (11)	Cl3—Ag3—S3—C91	72.71 (11)
C8—N3—C1—N1	177.1 (3)	C98—N13—C91—N11	179.3 (3)
C8—N3—C1—S1	−3.0 (4)	C98—N13—C91—S3	−0.4 (4)
N2—N1—C1—N3	7.9 (4)	N12—N11—C91—N13	−9.7 (4)
N2—N1—C1—S1	−172.1 (2)	N12—N11—C91—S3	170.0 (2)
Ag1—S1—C1—N3	−130.6 (3)	Ag3—S3—C91—N13	135.6 (2)
Ag1—S1—C1—N1	49.3 (3)	Ag3—S3—C91—N11	−44.0 (3)
N1—N2—C2—C4	−178.4 (3)	N11—N12—C92—C94	177.2 (3)
N1—N2—C2—C3	2.9 (5)	N11—N12—C92—C93	−2.0 (5)
C5—N4—C4—C7	4.2 (5)	C95—N14—C94—C97	−3.8 (5)
C5—N4—C4—C2	−174.9 (3)	C95—N14—C94—C92	177.1 (3)
N2—C2—C4—N4	166.8 (3)	N12—C92—C94—N14	−168.3 (3)
C3—C2—C4—N4	−14.5 (5)	C93—C92—C94—N14	11.0 (4)
N2—C2—C4—C7	−12.3 (5)	N12—C92—C94—C97	12.6 (4)
C3—C2—C4—C7	166.4 (3)	C93—C92—C94—C97	−168.2 (3)
C4—N4—C5—C6	−0.7 (6)	C94—N14—C95—C96	0.1 (5)
C7—N5—C6—C5	3.1 (5)	C97—N15—C96—C95	−1.9 (5)
N4—C5—C6—N5	−3.1 (6)	N14—C95—C96—N15	2.9 (6)
C6—N5—C7—C4	0.5 (5)	C96—N15—C97—C94	−1.8 (5)
N4—C4—C7—N5	−4.3 (5)	N14—C94—C97—N15	4.9 (5)
C2—C4—C7—N5	174.8 (3)	C92—C94—C97—N15	−176.1 (3)
C1—N3—C8—C9	−77.7 (4)	C91—N13—C98—C99	79.4 (4)
C16—P1—C10—C15	−32.8 (3)	C106—P5—C100—C105	136.8 (3)
C22—P1—C10—C15	75.4 (3)	C112—P5—C100—C105	−115.4 (3)
Ag1—P1—C10—C15	−160.8 (2)	Ag3—P5—C100—C105	4.2 (3)
C16—P1—C10—C11	149.4 (2)	C106—P5—C100—C101	−40.5 (3)
C22—P1—C10—C11	−102.3 (2)	C112—P5—C100—C101	67.3 (3)
Ag1—P1—C10—C11	21.5 (3)	Ag3—P5—C100—C101	−173.0 (2)
C15—C10—C11—C12	−2.0 (4)	C105—C100—C101—C102	2.5 (4)
P1—C10—C11—C12	175.7 (2)	P5—C100—C101—C102	179.7 (2)
C10—C11—C12—C13	−0.3 (4)	C100—C101—C102—C103	−0.8 (4)
C11—C12—C13—C14	2.1 (4)	C101—C102—C103—C104	−1.8 (5)
C12—C13—C14—C15	−1.5 (5)	C102—C103—C104—C105	2.6 (5)
C11—C10—C15—C14	2.6 (4)	C101—C100—C105—C104	−1.7 (5)
P1—C10—C15—C14	−175.1 (2)	P5—C100—C105—C104	−179.0 (3)
C13—C14—C15—C10	−0.9 (4)	C103—C104—C105—C100	−0.8 (6)
C10—P1—C16—C21	−84.9 (3)	C112—P5—C106—C107	179.9 (2)
C22—P1—C16—C21	167.7 (3)	C100—P5—C106—C107	−70.2 (2)
Ag1—P1—C16—C21	48.1 (3)	Ag3—P5—C106—C107	61.1 (3)
C10—P1—C16—C17	96.7 (3)	C112—P5—C106—C111	0.0 (3)
C22—P1—C16—C17	−10.8 (3)	C100—P5—C106—C111	109.9 (2)
Ag1—P1—C16—C17	−130.3 (2)	Ag3—P5—C106—C111	−118.8 (2)
C21—C16—C17—C18	1.5 (5)	C111—C106—C107—C108	−0.2 (4)
P1—C16—C17—C18	180.0 (2)	P5—C106—C107—C108	180.0 (2)
C16—C17—C18—C19	1.1 (5)	C106—C107—C108—C109	−1.3 (4)
C17—C18—C19—C20	−1.7 (5)	C107—C108—C109—C110	2.0 (4)
C18—C19—C20—C21	−0.4 (6)	C108—C109—C110—C111	−1.3 (4)
C17—C16—C21—C20	−3.6 (5)	C109—C110—C111—C106	−0.1 (4)
P1—C16—C21—C20	177.9 (3)	C107—C106—C111—C110	0.9 (4)
C19—C20—C21—C16	3.0 (6)	P5—C106—C111—C110	−179.3 (2)
C16—P1—C22—C27	−74.5 (3)	C106—P5—C112—C117	−92.7 (2)
C10—P1—C22—C27	178.3 (2)	C100—P5—C112—C117	161.3 (2)
Ag1—P1—C22—C27	47.3 (3)	Ag3—P5—C112—C117	34.8 (2)
C16—P1—C22—C23	108.1 (3)	C106—P5—C112—C113	87.2 (3)
C10—P1—C22—C23	0.9 (3)	C100—P5—C112—C113	−18.8 (3)
Ag1—P1—C22—C23	−130.1 (2)	Ag3—P5—C112—C113	−145.4 (2)
C27—C22—C23—C24	2.0 (5)	C117—C112—C113—C114	0.9 (4)
P1—C22—C23—C24	179.4 (3)	P5—C112—C113—C114	−179.0 (2)
C22—C23—C24—C25	−0.5 (5)	C112—C113—C114—C115	0.1 (5)
C23—C24—C25—C26	−0.2 (5)	C113—C114—C115—C116	−0.9 (5)
C24—C25—C26—C27	−0.6 (5)	C114—C115—C116—C117	0.7 (5)
C23—C22—C27—C26	−2.8 (5)	C113—C112—C117—C116	−1.1 (5)
P1—C22—C27—C26	179.6 (2)	P5—C112—C117—C116	178.8 (3)
C25—C26—C27—C22	2.2 (5)	C115—C116—C117—C112	0.3 (5)
C34—P2—C28—C29	17.2 (3)	C130—P6—C118—C123	101.4 (3)
C40—P2—C28—C29	−91.1 (3)	C124—P6—C118—C123	−4.9 (3)
Ag1—P2—C28—C29	144.6 (2)	Ag3—P6—C118—C123	−132.8 (2)
C34—P2—C28—C33	−162.8 (3)	C130—P6—C118—C119	−78.0 (3)
C40—P2—C28—C33	88.9 (3)	C124—P6—C118—C119	175.7 (2)
Ag1—P2—C28—C33	−35.4 (3)	Ag3—P6—C118—C119	47.9 (3)
C33—C28—C29—C30	−0.9 (5)	C123—C118—C119—C120	−2.3 (5)
P2—C28—C29—C30	179.1 (3)	P6—C118—C119—C120	177.1 (3)
C28—C29—C30—C31	−1.4 (5)	C118—C119—C120—C121	0.5 (5)
C29—C30—C31—C32	1.9 (5)	C119—C120—C121—C122	1.5 (5)
C30—C31—C32—C33	−0.2 (6)	C120—C121—C122—C123	−1.8 (5)
C31—C32—C33—C28	−2.1 (5)	C119—C118—C123—C122	2.0 (5)
C29—C28—C33—C32	2.6 (5)	P6—C118—C123—C122	−177.3 (3)
P2—C28—C33—C32	−177.4 (3)	C121—C122—C123—C118	0.0 (5)
C28—P2—C34—C35	112.0 (3)	C130—P6—C124—C125	−20.6 (3)
C40—P2—C34—C35	−140.5 (3)	C118—P6—C124—C125	86.7 (3)
Ag1—P2—C34—C35	−17.6 (3)	Ag3—P6—C124—C125	−143.5 (2)
C28—P2—C34—C39	−69.6 (3)	C130—P6—C124—C129	160.0 (2)
C40—P2—C34—C39	37.9 (3)	C118—P6—C124—C129	−92.7 (2)
Ag1—P2—C34—C39	160.8 (2)	Ag3—P6—C124—C129	37.1 (3)
C39—C34—C35—C36	−1.3 (5)	C129—C124—C125—C126	0.2 (4)
P2—C34—C35—C36	177.2 (3)	P6—C124—C125—C126	−179.2 (2)
C34—C35—C36—C37	0.9 (6)	C124—C125—C126—C127	0.8 (3)
C35—C36—C37—C38	−0.4 (5)	C125—C126—C127—C128	−1.1 (2)
C36—C37—C38—C39	0.4 (5)	C126—C127—C128—C129	0.4 (4)
C37—C38—C39—C34	−0.8 (5)	C125—C124—C129—C128	−1.0 (4)
C35—C34—C39—C38	1.2 (5)	P6—C124—C129—C128	178.5 (2)
P2—C34—C39—C38	−177.2 (2)	C127—C128—C129—C124	0.6 (5)
C34—P2—C40—C41	−150.4 (3)	C118—P6—C130—C131	2.2 (3)
C28—P2—C40—C41	−39.8 (3)	C124—P6—C130—C131	109.4 (3)
Ag1—P2—C40—C41	86.9 (3)	Ag3—P6—C130—C131	−126.6 (2)
C34—P2—C40—C45	35.7 (3)	C118—P6—C130—C135	−178.9 (2)
C28—P2—C40—C45	146.3 (3)	C124—P6—C130—C135	−71.8 (2)
Ag1—P2—C40—C45	−87.0 (3)	Ag3—P6—C130—C135	52.2 (2)
C45—C40—C41—C42	0.6 (5)	C135—C130—C131—C132	0.7 (5)
P2—C40—C41—C42	−173.4 (3)	P6—C130—C131—C132	179.6 (3)
C40—C41—C42—C43	−1.6 (6)	C130—C131—C132—C133	0.1 (5)
C41—C42—C43—C44	0.9 (6)	C131—C132—C133—C134	−0.8 (5)
C42—C43—C44—C45	0.9 (6)	C132—C133—C134—C135	0.6 (5)
C41—C40—C45—C44	1.2 (5)	C133—C134—C135—C130	0.3 (5)
P2—C40—C45—C44	175.2 (3)	C131—C130—C135—C134	−0.9 (4)
C43—C44—C45—C40	−2.0 (6)	P6—C130—C135—C134	−179.8 (2)
C46—N6—N7—C47	−176.4 (3)	C136—N16—N17—C137	171.8 (3)
P4—Ag2—P3—C61	−45.67 (12)	P8—Ag4—P7—C145	−170.74 (11)
Cl2—Ag2—P3—C61	99.11 (12)	Cl4—Ag4—P7—C145	44.22 (11)
S2—Ag2—P3—C61	−167.51 (12)	S4—Ag4—P7—C145	−49.88 (11)
P4—Ag2—P3—C55	71.71 (10)	P8—Ag4—P7—C157	65.57 (13)
Cl2—Ag2—P3—C55	−143.51 (10)	Cl4—Ag4—P7—C157	−79.47 (13)
S2—Ag2—P3—C55	−50.13 (10)	S4—Ag4—P7—C157	−173.58 (12)
P4—Ag2—P3—C67	−169.38 (11)	P8—Ag4—P7—C151	−55.65 (11)
Cl2—Ag2—P3—C67	−24.60 (12)	Cl4—Ag4—P7—C151	159.31 (10)
S2—Ag2—P3—C67	68.79 (11)	S4—Ag4—P7—C151	65.21 (11)
P3—Ag2—P4—C85	−71.76 (12)	P7—Ag4—P8—C163	−159.19 (12)
Cl2—Ag2—P4—C85	141.31 (12)	Cl4—Ag4—P8—C163	−13.55 (12)
S2—Ag2—P4—C85	40.90 (12)	S4—Ag4—P8—C163	85.09 (12)
P3—Ag2—P4—C73	166.14 (12)	P7—Ag4—P8—C169	−40.81 (13)
Cl2—Ag2—P4—C73	19.21 (12)	Cl4—Ag4—P8—C169	104.82 (13)
S2—Ag2—P4—C73	−81.19 (12)	S4—Ag4—P8—C169	−156.53 (13)
P3—Ag2—P4—C79	44.65 (11)	P7—Ag4—P8—C175	76.10 (12)
Cl2—Ag2—P4—C79	−102.28 (10)	Cl4—Ag4—P8—C175	−138.26 (12)
S2—Ag2—P4—C79	157.32 (10)	S4—Ag4—P8—C175	−39.62 (12)
P3—Ag2—S2—C46	167.73 (11)	P7—Ag4—S4—C136	−163.09 (11)
P4—Ag2—S2—C46	41.97 (11)	P8—Ag4—S4—C136	−40.74 (11)
Cl2—Ag2—S2—C46	−72.89 (11)	Cl4—Ag4—S4—C136	77.76 (11)
C53—N8—C46—N6	179.8 (3)	C143—N18—C136—N16	−172.0 (3)
C53—N8—C46—S2	−0.9 (4)	C143—N18—C136—S4	7.9 (5)
N7—N6—C46—N8	10.5 (4)	N17—N16—C136—N18	−7.2 (4)
N7—N6—C46—S2	−168.8 (2)	N17—N16—C136—S4	172.9 (2)
Ag2—S2—C46—N8	−136.1 (2)	Ag4—S4—C136—N18	133.4 (3)
Ag2—S2—C46—N6	43.2 (3)	Ag4—S4—C136—N16	−46.7 (3)
N6—N7—C47—C49	−177.6 (3)	N16—N17—C137—C139	176.8 (3)
N6—N7—C47—C48	2.1 (5)	N16—N17—C137—C138	−2.3 (5)
C50—N9—C49—C52	4.3 (5)	C140—N19—C139—C142	−4.9 (5)
C50—N9—C49—C47	−173.5 (3)	C140—N19—C139—C137	174.4 (3)
N7—C47—C49—N9	166.7 (3)	N17—C137—C139—N19	−163.8 (3)
C48—C47—C49—N9	−13.0 (4)	C138—C137—C139—N19	15.4 (4)
N7—C47—C49—C52	−11.2 (4)	N17—C137—C139—C142	15.5 (4)
C48—C47—C49—C52	169.2 (3)	C138—C137—C139—C142	−165.3 (3)
C49—N9—C50—C51	0.4 (5)	C139—N19—C140—C141	0.4 (6)
C52—N10—C51—C50	3.8 (5)	C142—N20—C141—C140	−2.5 (6)
N9—C50—C51—N10	−4.7 (6)	N19—C140—C141—N20	3.5 (7)
C51—N10—C52—C49	1.0 (5)	C141—N20—C142—C139	−2.1 (5)
N9—C49—C52—N10	−5.3 (5)	N19—C139—C142—N20	6.0 (5)
C47—C49—C52—N10	172.5 (3)	C137—C139—C142—N20	−173.3 (3)
C46—N8—C53—C54	−80.1 (4)	C136—N18—C143—C144	86.4 (4)
C61—P3—C55—C56	93.1 (3)	C157—P7—C145—C146	−90.1 (3)
C67—P3—C55—C56	−160.5 (2)	C151—P7—C145—C146	17.1 (3)
Ag2—P3—C55—C56	−35.1 (3)	Ag4—P7—C145—C146	135.4 (2)
C61—P3—C55—C60	−87.9 (3)	C157—P7—C145—C150	90.1 (3)
C67—P3—C55—C60	18.5 (3)	C151—P7—C145—C150	−162.6 (3)
Ag2—P3—C55—C60	143.9 (2)	Ag4—P7—C145—C150	−44.4 (3)
C60—C55—C56—C57	2.1 (5)	C150—C145—C146—C147	−2.4 (5)
P3—C55—C56—C57	−178.8 (3)	P7—C145—C146—C147	177.8 (2)
C55—C56—C57—C58	−0.8 (5)	C145—C146—C147—C148	−0.1 (5)
C56—C57—C58—C59	−0.8 (5)	C146—C147—C148—C149	1.5 (5)
C57—C58—C59—C60	1.1 (5)	C147—C148—C149—C150	−0.4 (6)
C58—C59—C60—C55	0.2 (5)	C148—C149—C150—C145	−2.2 (6)
C56—C55—C60—C59	−1.8 (4)	C146—C145—C150—C149	3.6 (5)
P3—C55—C60—C59	179.3 (2)	P7—C145—C150—C149	−176.7 (3)
C55—P3—C61—C66	−179.7 (2)	C145—P7—C151—C152	78.6 (3)
C67—P3—C61—C66	70.3 (2)	C157—P7—C151—C152	−174.3 (2)
Ag2—P3—C61—C66	−60.4 (2)	Ag4—P7—C151—C152	−41.4 (2)
C55—P3—C61—C62	−1.2 (3)	C145—P7—C151—C156	−103.5 (3)
C67—P3—C61—C62	−111.3 (2)	C157—P7—C151—C156	3.7 (3)
Ag2—P3—C61—C62	118.0 (2)	Ag4—P7—C151—C156	136.6 (2)
C66—C61—C62—C63	−1.6 (4)	C156—C151—C152—C153	1.5 (4)
P3—C61—C62—C63	−180.0 (2)	P7—C151—C152—C153	179.6 (2)
C61—C62—C63—C64	0.5 (4)	C151—C152—C153—C154	−0.7 (5)
C62—C63—C64—C65	1.0 (4)	C152—C153—C154—C155	−0.3 (5)
C63—C64—C65—C66	−1.4 (4)	C153—C154—C155—C156	0.3 (5)
C62—C61—C66—C65	1.2 (4)	C154—C155—C156—C151	0.6 (5)
P3—C61—C66—C65	179.7 (2)	C152—C151—C156—C155	−1.5 (5)
C64—C65—C66—C61	0.3 (4)	P7—C151—C156—C155	−179.5 (2)
C61—P3—C67—C72	−136.3 (3)	C145—P7—C157—C162	−149.3 (2)
C55—P3—C67—C72	116.0 (3)	C151—P7—C157—C162	102.5 (2)
Ag2—P3—C67—C72	−3.3 (3)	Ag4—P7—C157—C162	−21.6 (3)
C61—P3—C67—C68	40.0 (3)	C145—P7—C157—C158	33.4 (3)
C55—P3—C67—C68	−67.6 (3)	C151—P7—C157—C158	−74.8 (3)
Ag2—P3—C67—C68	173.1 (2)	Ag4—P7—C157—C158	161.1 (2)
C72—C67—C68—C69	−3.6 (4)	C162—C157—C158—C159	−3.2 (4)
P3—C67—C68—C69	−179.9 (2)	P7—C157—C158—C159	174.2 (2)
C67—C68—C69—C70	1.6 (4)	C157—C158—C159—C160	1.3 (4)
C68—C69—C70—C71	0.8 (5)	C158—C159—C160—C161	1.2 (4)
C69—C70—C71—C72	−1.2 (5)	C159—C160—C161—C162	−1.8 (5)
C68—C67—C72—C71	3.1 (5)	C158—C157—C162—C161	2.6 (4)
P3—C67—C72—C71	179.5 (3)	P7—C157—C162—C161	−174.8 (2)
C70—C71—C72—C67	−0.7 (5)	C160—C161—C162—C157	−0.2 (4)
C85—P4—C73—C74	−174.1 (3)	C169—P8—C163—C164	−88.6 (3)
C79—P4—C73—C74	79.4 (3)	C175—P8—C163—C164	163.0 (3)
Ag2—P4—C73—C74	−46.8 (3)	Ag4—P8—C163—C164	34.8 (3)
C85—P4—C73—C78	6.1 (3)	C169—P8—C163—C168	90.5 (3)
C79—P4—C73—C78	−100.4 (3)	C175—P8—C163—C168	−17.9 (3)
Ag2—P4—C73—C78	133.4 (2)	Ag4—P8—C163—C168	−146.1 (2)
C78—C73—C74—C75	3.1 (5)	C168—C163—C164—C165	−1.8 (5)
P4—C73—C74—C75	−176.7 (3)	P8—C163—C164—C165	177.4 (3)
C73—C74—C75—C76	−2.5 (5)	C163—C164—C165—C166	1.7 (5)
C74—C75—C76—C77	0.1 (5)	C164—C165—C166—C167	−0.5 (5)
C75—C76—C77—C78	1.7 (5)	C165—C166—C167—C168	−0.5 (5)
C76—C77—C78—C73	−1.1 (5)	C164—C163—C168—C167	0.8 (5)
C74—C73—C78—C77	−1.3 (5)	P8—C163—C168—C167	−178.3 (3)
P4—C73—C78—C77	178.5 (2)	C166—C167—C168—C163	0.3 (5)
C85—P4—C79—C80	−109.9 (3)	C163—P8—C169—C174	36.9 (3)
C73—P4—C79—C80	−2.4 (3)	C175—P8—C169—C174	147.6 (3)
Ag2—P4—C79—C80	127.0 (2)	Ag4—P8—C169—C174	−89.6 (3)
C85—P4—C79—C84	71.8 (2)	C163—P8—C169—C170	−148.7 (3)
C73—P4—C79—C84	179.3 (2)	C175—P8—C169—C170	−38.0 (3)
Ag2—P4—C79—C84	−51.3 (2)	Ag4—P8—C169—C170	84.8 (3)
C84—C79—C80—C81	−0.2 (4)	C174—C169—C170—C171	−1.6 (5)
P4—C79—C80—C81	−178.5 (2)	P8—C169—C170—C171	−176.2 (3)
C79—C80—C81—C82	−1.2 (5)	C169—C170—C171—C172	2.4 (6)
C80—C81—C82—C83	1.6 (5)	C170—C171—C172—C173	−2.2 (7)
C81—C82—C83—C84	−0.5 (5)	C171—C172—C173—C174	1.1 (7)
C82—C83—C84—C79	−1.0 (5)	C170—C169—C174—C173	0.6 (5)
C80—C79—C84—C83	1.3 (5)	P8—C169—C174—C173	175.1 (3)
P4—C79—C84—C83	179.7 (3)	C172—C173—C174—C169	−0.4 (6)
C73—P4—C85—C90	−84.4 (3)	C163—P8—C175—C176	−109.5 (3)
C79—P4—C85—C90	23.2 (3)	C169—P8—C175—C176	142.6 (3)
Ag2—P4—C85—C90	145.8 (2)	Ag4—P8—C175—C176	20.7 (3)
C73—P4—C85—C86	95.2 (2)	C163—P8—C175—C180	73.0 (3)
C79—P4—C85—C86	−157.3 (2)	C169—P8—C175—C180	−34.9 (3)
Ag2—P4—C85—C86	−34.6 (3)	Ag4—P8—C175—C180	−156.8 (2)
C90—C85—C86—C87	−0.4 (4)	C180—C175—C176—C177	0.0 (5)
P4—C85—C86—C87	−179.9 (2)	P8—C175—C176—C177	−177.6 (3)
C85—C86—C87—C88	0.6 (5)	C175—C176—C177—C178	1.2 (5)
C86—C87—C88—C89	−0.6 (5)	C176—C177—C178—C179	−1.9 (5)
C87—C88—C89—C90	0.4 (5)	C177—C178—C179—C180	1.4 (5)
C88—C89—C90—C85	−0.2 (5)	C178—C179—C180—C175	−0.1 (5)
C86—C85—C90—C89	0.2 (4)	C176—C175—C180—C179	−0.6 (5)
P4—C85—C90—C89	179.8 (2)	P8—C175—C180—C179	176.9 (2)

### Hydrogen-bond geometry (Å, º)

**Table:**

*D*—H···*A*	*D*—H	H···*A*	*D*···*A*	*D*—H···*A*
N1—H1*N*···Cl1	0.88 (2)	2.54 (2)	3.381 (3)	163 (3)
N3—H3*N*···Cl3	0.87 (2)	2.68 (3)	3.406 (3)	142 (3)
N6—H6*N*···Cl2	0.88 (2)	2.53 (2)	3.370 (3)	162 (3)
N8—H8*N*···Cl4	0.86 (2)	2.68 (3)	3.411 (3)	143 (3)
N11—H11*N*···Cl3	0.88 (2)	2.52 (2)	3.363 (3)	161 (3)
N13—H13*N*···Cl2	0.88 (2)	2.65 (2)	3.423 (3)	147 (3)
N16—H16*N*···Cl4	0.86 (2)	2.50 (2)	3.338 (3)	164 (3)
N18—H18*N*···Cl1^i^	0.86 (2)	2.70 (3)	3.425 (3)	143 (3)

Symmetry code: (i) *x*−1, *y*, *z*−1.
